# Whole‐Genome Sequence of Synthesized Allopolyploids in *Cucumis* Reveals Insights into the Genome Evolution of Allopolyploidization

**DOI:** 10.1002/advs.202004222

**Published:** 2021-02-15

**Authors:** Xiaqing Yu, Panqiao Wang, Ji Li, Qinzheng Zhao, Changmian Ji, Zaobing Zhu, Yufei Zhai, Xiaodong Qin, Junguo Zhou, Haiyan Yu, Xinchao Cheng, Shiro Isshiki, Molly Jahn, Jeff J. Doyle, Carl‐Otto Ottosen, Yuling Bai, Qinsheng Cai, Chunyan Cheng, Qunfeng Lou, Sanwen Huang, Jinfeng Chen

**Affiliations:** ^1^ National Key Laboratory of Crop Genetics and Germplasm Enhancement Nanjing Agricultural University Nanjing 210095 China; ^2^ Hainan Key Laboratory for Biosafety Monitoring and Molecular Breeding in Off‐Season Reproduction Regions Institute of Tropical Bioscience and Biotechnology Chinese Academy of Tropical Agricultural Sciences Haikou 571101 China; ^3^ Biomarker Technologies Beijing 101300 China; ^4^ College of Horticulture and Landscape Henan Institute of Science and Technology Xinxiang 453000 China; ^5^ Faculty of Agriculture Saga University Saga 840‐8502 Japan; ^6^ Jahn Research Group USDA/FPL Madison WI 53726 USA; ^7^ Section of Plant Breeding and Genetics School of Integrated Plant Sciences Cornell University Ithaca NY 14853 USA; ^8^ Department of Food Science Aarhus University Aarhus 8200 Denmark; ^9^ Department of Plant Sciences Wageningen University and Research Wageningen 6700 AJ Netherlands; ^10^ College of Life Science Nanjing Agricultural University Nanjing 210095 China; ^11^ Agricultural Genomics Institute at Shenzhen Chinese Academy of Agricultural Sciences Shenzhen 518124 China

**Keywords:** allopolyploidy, *Cucumis*, diploidization, evolution, genomes

## Abstract

The importance of allopolyploidy in plant evolution has been widely recognized. The genetic changes triggered by allopolyploidy, however, are not yet fully understood due to inconsistent phenomena reported across diverse species. The construction of synthetic polyploids offers a controlled approach to systematically reveal genomic changes that occur during the process of polyploidy. This study reports the first fully sequenced synthetic allopolyploid constructed from a cross between *Cucumis sativus* and *C. hystrix*, with high‐quality assembly. The two subgenomes are confidently partitioned and the *C. sativus*‐originated subgenome predominates over the *C. hystrix*‐originated subgenome, retaining more sequences and showing higher homeologous gene expression. Most of the genomic changes emerge immediately after interspecific hybridization. Analysis of a series of genome sequences from several generations (S_0_, S_4_–S_13_) of *C*. ×*hytivus* confirms that genomic changes occurred in the very first generations, subsequently slowing down as the process of diploidization is initiated. The duplicated genome of the allopolyploid with double genes from both parents broadens the genetic base of *C*. ×*hytivus*, resulting in enhanced phenotypic plasticity. This study provides novel insights into plant polyploid genome evolution and demonstrates a promising strategy for the development of a wide array of novel plant species and varieties through artificial polyploidization.

## Graphical abstract

The first whole‐genome sequence of a synthetic allopolyploid in *Cucumis* demonstrates significant subgenome dominance. Comparative genomic analyses on interspecific hybrid and different generations of allopolyploids successfully differentiate the divergent roles of hybridization, genome duplication, and diploidization in shaping the genome. Moreover, the merger of two distinct genomes practically creates a novel allopolyploid species with higher fitness.

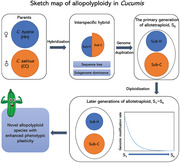

## 1 Introduction

Polyploids are organisms that contain three or more sets of chromosomes. They are mainly grouped into two types, autopolyploid and allopolyploid, depending on whether the multiple chromosome sets are identical or divergent. The prevalence of polyploids in nature demonstrates the evolutionary importance of polyploidy.^[^ ^1^ ^]^ The success of allopolyploids suggests their evolutionary advantage owing to their increased diversity and plasticity.^[^ ^2^ ^]^ However, allopolyploids face the challenge of coordinating distinct subgenomes with independent genetics and epigenetics into a single nucleus.^[^ ^3^ ^]^ The merger of two or more divergent genomes is believed to cause “genomic shock” in the newly formed allopolyploid, resulting in genome‐wide changes of gene structure and expression.^[^ ^4^ ^]^ One of the subgenomes may become dominant over other subgenome(s) experiencing less sequence loss and higher homeologous gene expression.^[^ ^5^ ^]^ In other instances, allopolyploids do not show subgenome dominance, for example, *Cucurbita*.^[^ ^6^ ^]^ Recent studies have suggested that the abundance and distribution of transposable elements (TEs) play a decisive role in this dominance.^[^ ^6^, ^7^ ^]^ These genomic changes are associated with phenotypic variation in allopolyploids,^[^ ^8^ ^]^ which may ultimately contribute to their establishment and survival in nature.^[^ ^9^ ^]^ Natural polyploids do not offer a system to study the mechanism by which cohabiting genomes are established. The exact parental genomes are often unknown or have evolved substantially since polyploid formation, and it is impossible to separately investigate changes resulting from the distinct events of interspecific hybridization and genome duplication. Efforts have been made for decades in different polyploid systems, yet the combined processes of hybridization and duplication when analyzed as snapshots seem to generate a range of possible responses that vary among genera. The examination of a synthetic allopolyploid with a defined genetic background and clear genetic history could reveal the underlying mechanisms for the distinct processes that occur during polyploidization.^[^ ^10^ ^]^ Molecular genetics and genomic approaches applied to a synthetic allopolyploid and its derived genotypes allow a first glimpse at the process that accounts for the widespread radiation of allopolyploids in nature and agriculture.

During domestication, crops undergo evolutionary bottlenecks where genetic diversity is rapidly lost relative to wild populations; cucumber is a good example.^[^ ^11^ ^]^ Cucumber (*Cucumis sativus* L.) (2*n* = 2*x* = 14), an economically important vegetable crop all over the world, is especially narrow in its genetic base.^[^ ^11^, ^12^ ^]^ Compared to other species, cucumber showed significantly fewer (61) nucleotide‐binding site (NBS) containing resistance genes,^[^ ^13^ ^]^ highlighting the opportunity to test new approaches to enhance genetic diversity.

*C. hystrix* Chakr. (2*n* = 2*x* = 24) is a wild Asiatic species, rediscovered in an isolated forest in the early 1990s that is possible to cross with cucumber, although with great difficulty.^[^ ^14^ ^]^ A synthetic allotetraploid species, *C*. × *hytivus* Chen and Kirkbride (2*n* = 4*x* = 38; containing both diploid genomes designated HHCC), was obtained via an interspecific hybridization between *C*. *hystrix* (2*n* = 2*x* = 24; HH) and *C*. *sativus* (2*n* = 2*x* = 14; CC) followed by genome duplication.^[^ ^15^ ^]^ This interspecific amphidiploid defined a new species with fixed heterozygosity with the further possibility of introducing a wide array of novel and potentially useful genes into cucumber via sexual hybridization. Indeed, introgression lines derived from backcrossing the amphidiploid with cucumber showed increased genetic diversity, including vigorous vegetative growth, higher yield,^[^ ^12b^ ^]^ and improved resistance against several diseases, including powdery mildew,^[^ ^16^ ^]^ downy mildew, and root‐knot nematode (RKN).^[^ ^17^ ^]^

Moreover, this amphidiploid, *C*. *×hytivus*, can also be used as a model system to explore the process of allopolyploidization. Our previous studies have shown both genetic and epigenetic reprogramming in *C*. *×hytivus*, which may contribute to the novel phenotypic variation found in amphidiploids, such as delayed leaf maturation.^[^ ^18^ ^]^ However, understanding the underlying mechanisms has been limited by the lack of genomic information about this synthetic species. In the present study, several advanced technologies, including whole‐genome shotgun sequencing, single‐molecule real‐time (SMRT) sequencing, high‐throughput chromosome conformation capture (Hi‐C) technology, and BioNano optical genome mapping, were adopted to generate a high‐quality genome sequence of *C*. *×hytivus*. Additionally, genome assembly of the unduplicated F_1_ homoploid hybrid and several early generations (S_0_, S_4_–S_13_) of *C*. *×hytivus* were obtained through shotgun sequencing to differentiate the genomic consequences of interspecific hybridization from the genomic consequence of genome duplication. Furthermore, we systematically examined individuals drawn from repeated rounds of self‐pollination to reveal the genomic changes that occur after formation of the amphidiploid. By sequencing individuals that essentially define a time series through fourteen generations of inbreeding, we reveal the genomic basis for the phenomenon of “diploidization” observed in allotetraploid.

## 2 Results

### 2.1 Assembly and Annotation of the *C*. ×*hytivus* Synthetic Allotetraploid Genome

**Figure 1:**
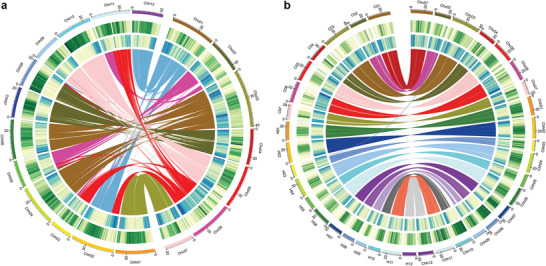
Characterization of the *C*. ×*hytivus* genome and chromosomes. a) Circos diagram showing relationships of Chc and Chh subgenome chromosomal pseudomolecules. The scale for the chromosomes (outer bars) is megabase; colors represent the density of transposon elements (blue) and genes (green). Homeologous blocks of ≥30 gene pairs between Chc01–Chc07 and Chh01–Chh12 are connected with lines. b) Syntenic comparisons between *C*. ×*hytivus* subgenomes and diploid HH and CC genomes. The outer three circles are chromosomes, density of genes, and density of transposon elements, respectively. Colored lines connect blocks with ≥30 orthologous gene pairs between the Chc and Chh subgenomes and CC and HH genomes, respectively, based on BLASP.

We developed a high‐quality reference genome assembly for *C*. ×*hytivus* Chen and Kirkbride (14th self‐pollinated generation, S_14_) (Figure S1, Supporting Information). The assembly of 69‐fold PacBio single‐molecule long reads yielded contigs totaling 530.78 Mb with an N50 of 6.9 Mb (**Table** **1**). We also collected 730 970 BioNano DNA molecules over 100 kb, corresponding to 200 equivalents of the genome (Table S1, Supporting Information). The genome map assembled de novo consisted of 499 constituent genome maps with an average length of 1.66 Mb and N50 of 2.59 Mb. These assemblies were used to correct the PacBio genome assembly.^[^ ^19^ ^]^ The final assembly via the BioNano approach contains 596 scaffolds, with a scaffold N50 of 8.09 Mb (Table 1). The total assembly size of 540.74 Mb was ≈67% and ≈77% of the genome size estimated via flow cytometry and *K*‐mer depth distribution of sequenced reads, respectively (Figure S2 and Table S2, Supporting Information). By aligning all the Illumina short reads of *C*. ×*hytivus* (S_14_) against each type of repeat, we estimated the proportion of repeats to be 62.68%, whereas the assembled repeat proportion is 39.54% of the estimated genome size (699.87 Mb) (Table S3, Supporting Information), suggesting that the remaining unassembled genome (≈23%) was mostly repeat sequences that were abnormally deeply covered by Illumina reads (Figure S3, Supporting Information). Of these repeats, 10.33% were tandem repeat sequences (i.e., types I, II, III, IV satellite DNAs, 5S, and 45S rDNA) and 12.81% were other repeats (i.e., microsatellites, minisatellites, and unassembled interspersed repeats). We further anchored the genome to chromosome scale using Hi‐C data (104‐fold coverage) (Table S4, Supporting Information). Finally, a total length of 525.78 Mb was distributed across 19 pseudomolecules (Figure S4 and Table S5, Supporting Information), representing 97.23% of the assembly above. Of this, 490.71 Mb (93.33%) can be ordered and orientated (Table S5, Supporting Information). We designated chromosomes as Chc01–Chc07 and Chh01–Chh12, corresponding to C01–C07 and H01–H12 of the diploid *C. sativus* (CC) and *C. hystrix* (HH) chromosomes, respectively (**Figure** **1**).

**Table 1:** *C. *×*hytivus* (S_14_) reference genome assembly statistics

	PacBio	PaBio+BioNano	PaBio+BioNano+Hi‐C
Total assembly size of contigs [bp]	530 781 911	530 854 507	530 844 507
Number of contigs	716	716	771
N50 contig length [bp]	6 900 133	6 900 743	6 596 157
N90 contig length [bp]	756 312	756 360	657 835
L50 contig count	27	27	29
L90 contig count	112	112	121
Longest contig [bp]	26 058 674	26 071 117	26 071 117
Total assembly size of scaffolds [bp]	–	540 738 094	540 748 294
Number of scaffolds	–	596	562
N50 scaffold length [bp]	–	8 092 476	27 207 877
N90 scaffold length [bp]	–	1 500 330	15 854 818
L50 scaffold count	–	19	9
L90 scaffold count	–	74	19
Gap length	–	9 893 587	9 903 787
Missing bases [%]^A^	–	0.83%	1.83%

^A)^ Missing bases (%) = gap length/total assembly size × 100.

We identified 275.69 Mb of repetitive sequences in *C. ×hytivus* (S_14_), accounting for 50.98% of the assembly (Table S6, Supporting Information). Long terminal repeats comprise the majority of TEs, as in other sequenced *Cucumis* genomes.^[^ ^13^, ^20^, ^21^ ^]^ By partitioning the TEs into two subgenomes, we would be able to reveal changes in repeats after allopolyploidization in these two subgenomes in comparison with their parental repeats (Figure S5, Supporting Information). In general, the Chc subgenome (SubC) of *C. ×hytivus* (S_14_) contained less TEs than the diploid *C. sativus* (CC) genome, while the Chh subgenome (SubH) maintained almost the same content and proportion of different types of TEs.

**Figure 2:**
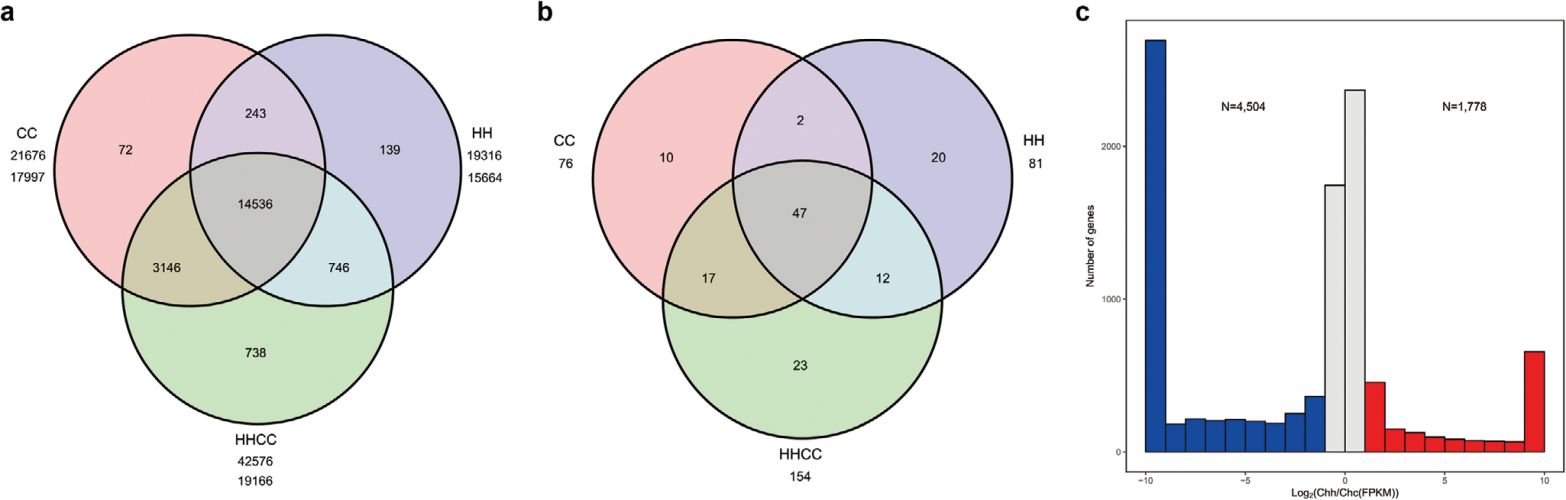
Changes of genes after allotetraploidization. a) Numbers of shared and unique orthologous protein‐coding gene clusters in *C. ×hytivus*, *C. hystrix*, and *C. sativus*. b) Numbers of shared and unique orthologous NBS‐encoding genes in *C. ×hytivus*, *C. hystrix*, and *C. sativus*. c) Histograms of genome‐wide expression of syntenic homeologous genes in *C. ×hytivus* (S_14_) leaves. *N* values indicate the total number of CC‐dominant (blue) and HH‐dominant (red) genes.

We used four gene‐prediction methods (RNA‐Seq, PacBio isoform sequencing (Iso‐Seq, Table S7, Supporting Information), homology‐based, and ab initio) to identify protein‐coding genes. A consensus gene set was constructed by merging all the results (Figure S6 and Table S8, Supporting Information). A total of 45 687 genes were predicted, with an average gene length of 3846 bp and 5.26 exons per gene (Table S9, Supporting Information). Approximately 97.53% of predicted genes could be annotated by matches with non‐redundant nucleotide and protein sequences in the The National Center for Biotechnology Information (NCBI), Cluster of Orthologous Groups, Gene Ontology (GO), Swiss‐Prot, and Kyoto Encyclopedia of Genes and Genomes (KEGG) databases (Table S10, Supporting Information). Genes are sparse near centromeric heterochromatin and abundant in distal euchromatin (Figure 1a). Identification of 90.90% of the 1440 genes in the Plantae Benchmarking Universal Single‐Copy Orthologs (BUSCO) dataset^[^ ^22^ ^]^ and 97.82% of 458 core eukaryotic genes (Cluster of Essential Genes database)^[^ ^23^ ^]^ indicated high‐quality genome assembly and annotation (Table S11, Supporting Information). Of the 18 882 orthologous gene families identified in CC and HH diploid genomes, 18 428 (97.60%) were also identified in the *C. ×hytivus* (S_14_) allotetraploid (**Figure** **2**). Additionally, we identified noncoding RNAs, including 134 microRNAs, 1274 tRNAs, 2125 rRNAs, and 573 small nuclear RNAs from the *C. ×hytivus* (S_14_) genome (Table S12, Supporting Information).

### 2.2 Subgenome Dominance

We divided the assembly of *C. ×hytivus* (S_14_) genome into Chc (203.36 Mb) and Chh (287.37 Mb) subgenomes, both of which are smaller than the corresponding CC genome (226.21 Mb)^[^ ^20^ ^]^ and the HH genome (297.49 Mb). Similarly, the Chc and Chh subgenomes contain 23 108 and 22 535 genes, respectively, which are less than the corresponding parental species, *C. sativus* (24 317) and *C. hystrix* (23 864) (Table S9, Supporting Information). This observation contrasts with previously published work in peanut,^[^ ^24^ ^]^ and suggests that the reported gene expansion after polyploidization in that species is not an inevitable result of polyploidization but could have occurred later in the process of diploidization. Nevertheless, the Chc and Chh subgenomes are largely colinear with the corresponding diploid parent HH and CC genomes, as shown by syntenic comparisons, which are mostly collinear (Figure 1b; Figure S7, Supporting Information). The colinearity of Chc04 with Chh07 and Chh08 was confirmed by cytological observation (Figure S8, Supporting Information). We used a previously synthesized oligo library of CC chromosome C04, which contains all oligos selected based on single copy sequences, to paint the pachytene chromosomes of *C. ×hytivus* (S_14_) by fluorescence in situ hybridization (FISH), employing a recently developed multiplex PCR‐based chromosome segmentation painting strategy^[^ ^25^ ^]^ with the caveat that oligo‐painting cannot detect repeated sequence changes and gene loss.

Although the differences in the accuracy of the parental genome assemblies may lead to biased results, analysis of gene colinearity in *C. ×hytivus* (S_14_) revealed that the CC genome was less fractionated than the HH genome (Figure S7a, Supporting Information). In addition, structural variant (SV) analysis using the actual parental genome reads also showed that more SVs were detected in the Chh subgenome than in the Chc subgenome (Table S13, Supporting Information). We also detected parental gene loss in *C. ×hytivus* (S_14_). We identified 21 382 (88% of CC genes) orthologous gene pairs between the Chc subgenome and CC parental genome, and 18 105 (76% of HH genes) orthologous gene pairs between Chh subgenome and HH parental genome (Tables S14 and S15, Supporting Information). Compared with CC, more HH genes appeared to be lost in *C. ×hytivus* (S_14_), although most of the orthologous gene pairs in CC and HH remained as homeologous pairs in *C. ×hytivus* (S_14_). Validation via sequence depth analysis confirmed the absence of 11 CC and 146 HH genes in the *C. ×hytivus* (S_14_) genome (Tables S16 and S17, Supporting Information). These observations indicate that the Chc subgenome may be dominant over that of the Chh subgenome.

Homoeologous exchange (HE) analysis demonstrated that more HH sequences were converted by CC sequences, consistent with the dominance of the CC genome (Tables S18 and S19, Supporting Information). Moreover, we investigated the expression of syntenic gene pairs in the subgenomes of *C. ×hytivus* (S_14_). The results revealed that CC‐dominant genes were expressed significantly more than HH‐dominant genes (Figure 2c; Table S20, Supporting Information), which again proved the dominance of the Chc subgenome. Our results reinforce the phenomenon of fractionation bias^[^ ^26^ ^]^ in allopolyploids. Fractionation bias is hypothesized to be driven by differential density of TE insertions in the progenitor genomes.^[^ ^27^ ^]^ In this model, inactivation of TEs spreads to nearby genes, such that, on average, the homoeologous genome with the greatest density of TEs has overall weaker expression, leading to a greater probability of gene inactivation and eventual loss.^[^ ^28^ ^]^ The genomes of the CC and Chc subgenomes contain fewer TEs than the HH genome and Chh subgenome (Figure S5, Supporting Information). Our study proved this hypothesis, showing that the overall TE densities near genes were lower for the Chc subgenome than for the other parental subgenome (Figure S9, Supporting Information).

### 2.3 Changes in Hybridization, Duplication, and Diploidization

**Figure 3:**
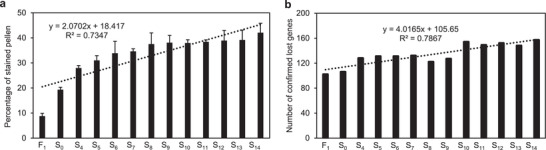
a) Pollen stainability of F_1_ homoploid hybrid and early generations of *C. ×hytivus*. Five biological replicates of 15 male flowers randomly collected from each generation of allotetraploid *C. ×hytivus* were assayed for pollen stainability (mean ± 5 SD). A minimum of 2000 pollen grains were collected for each biological replicate. b) Number of missing genes in F_1_ homoploid hybrid and early generations of *C. ×hytivus*.

**Figure 4:**
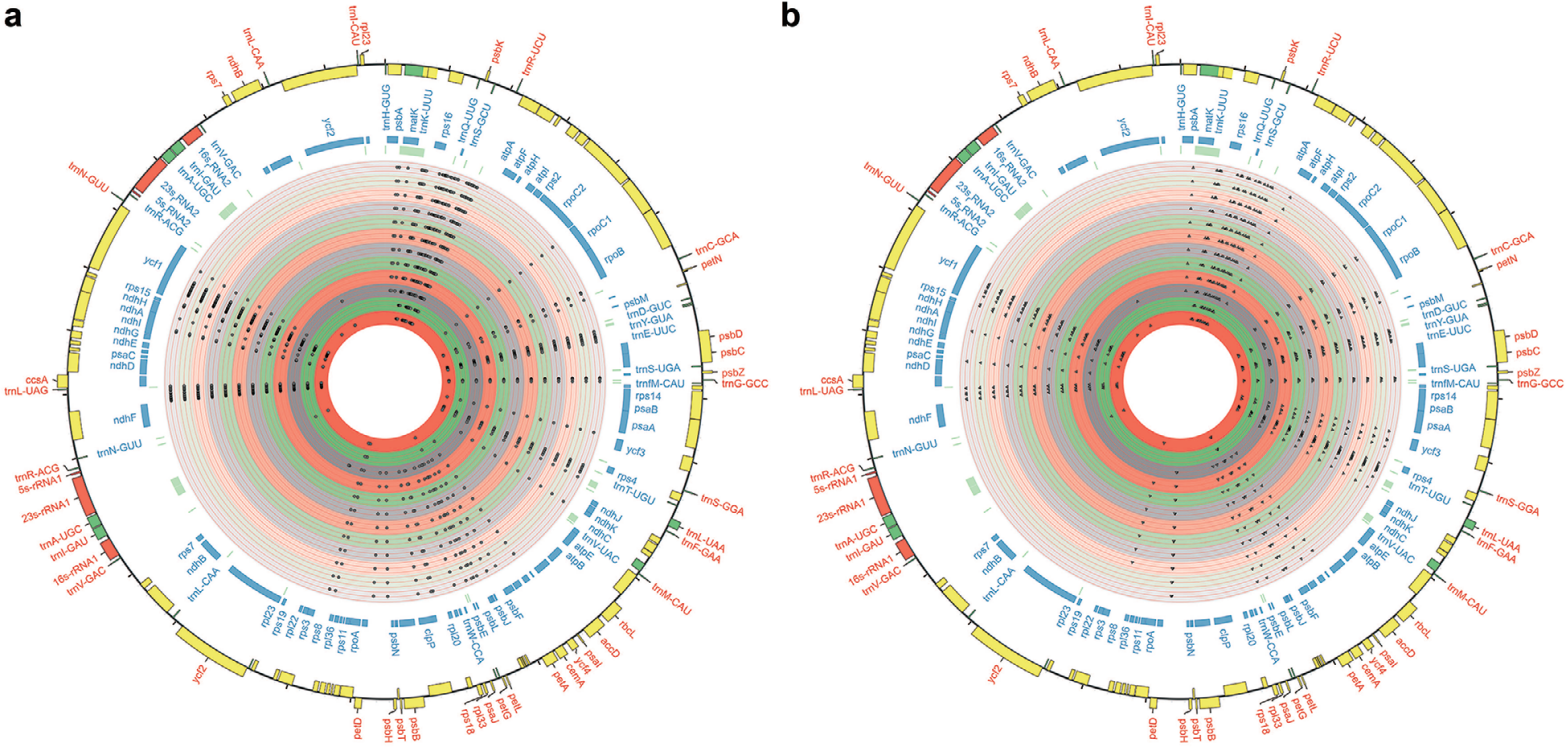
a) SNPs (closed circles) and b) indels (closed triangles) distribution of the cp genome of F_1_ homoploid hybrid and early generations of *C. ×hytivus* (from inner to outer circles) along the cp genome of *C. hystrix*.

To distinguish the effect of hybridization, duplication, and diploidization, we further identified 157 confirmed missing genes in the F_1_, S_0_, and other subsequent generations (S_4_–S_13_) using clean sequence read coverage analysis (Table S21, Supporting Information). The results showed that 102 genes were absent in F_1_ (**Figure** **3**b, Tables S22 and S23, Supporting Information), suggesting that nuclear sequence elimination occurred immediately after the interspecific hybridization event. More interestingly, few missing genes in F_1_ reappeared in S_0_ (Tables S22 and S23, Supporting Information), suggesting a distinct role of genome duplication from genome merger in allopolyploidy. The chloroplast (cp) genome is maternally inherited in *Cucumis* species.^[^ ^29^ ^]^ Comparative DNA sequence analyses revealed a total of 195 single nucleotide polymorphisms (SNPs) and 100 insertion–deletion polymorphisms (indels) in the cp genome in the F_1_ homoploid hybrid and early generations (S_0_, S_4_–S_13_) of *C*. *×hytivus* relative to the cp genome of HH (**Figure** **4**; Tables S24 and S25, Supporting Information). Of these, the majority of SNPs (73.85%) and indels (73%) were detected in F_1_, indicating a significantly larger effect of hybridization on the cp genome than as a result of duplication and diploidization during the process of allopolyploidization in *Cucumis*.

According to our results, the process of diploidization can be resolved into two distinct stages. The first stage is the first three generations (S_0_–S_4_), during which the changes are dramatic. Most nuclear and cp genomic changes that occurred after allopolyploidization were detected in S_4_ and inherited in later generations of *C. ×hytivus* (Figures 3b and 4; Tables S22–S25, Supporting Information), providing direct evidence that rapid genomic changes occurred in the first few generations after allopolyploidization. Accordingly, pollen viability increased by 9% (Figure 3a).

The second stage begins with the fourth and subsequent generation (S_4_–S_14_) where we observed only sporadic nuclear sequence loss (gene loss), and no new SNPs or indels in cp genomes were identified. Occasionally, anomalies of sequence loss (gene loss), SNPs, and indels were observed in different generations (Tables S22–S25, Supporting Information), which can be explained by the individual differences within each generation as only one plant was randomly chosen for sequencing. To investigate the meiotic behavior between two subgenomes during the process of diploidization, we performed genomic in situ hybridization (GISH) experiments on pollen mother cells (PMCs) at metaphase I (MI) and anaphase I (AI) of different generations of *C. ×hytivus*. Abnormal meiotic chromosome behaviors were frequently observed, including asynchronous meiosis at MI (Figure S10b, Supporting Information, white and red arrows), univalents (Figure S10c, Supporting Information), intergenomic pairings (Figure S10d,e, Supporting Information), and lagging chromosomes (Figure S10f, Supporting Information). Meiotic chromosome behaviors of six generations of plants (S_4_, S_6_, S_8_, S_10_, S_12_, and S_14_) showed that the number of univalents and lagging frequency of the Chc subgenome was significantly lower than that of the Chh subgenome (**Table** **2**). This suggests some instability of the Chh subgenome in this allotetraploid, which could potentially exhibit higher rates of lost sequence through inbreeding.

**Table 2:** Meiotic chromosome behavior in six different generations of the synthetic allotetraploid *C. ×hytivus*. Different lower case letters indicate significant difference between the values in each column by Duncan's test, *p* < 0.05

Generation	No. of PMCs^A^ at MI^B^	No. [%] of PMCs with 19 homologous bivalents	Bivalents [mean ± SD]		No. [%] of PMCs with univalent		No. [%] of PMCs with intergenomic pairings	No. of PMCs at AI^C^	No. [%] of PMCs with lagging chromosome	
			Chc	Chh	Chc	Chh			Chc	Chh
S_4_	143	49 (34.2)^e^	6.69 ± 0.5	10.81 ± 1.1	34 (23.5)^a^	93 (65.2)^a^	35 (24.4)^a^	95	26 (26.6)^a^	75 (79.0)^a^
S_6_	122	45 (36.9)^e^	6.71 ± 0.5	10.94 ± 1.0	24 (19.6)^b^	76 (62.5)^a^	24 (19.8)^b^	102	25 (24.3)^a,b^	74 (72.2)^a,b^
S_8_	104	44 (42.2)^d^	6.76 ± 0.5	11.13 ± 0.9	17 (16.6)^b^	57 (55.3)^b^	18 (17.1)^b,c^	89	18 (20.6)^a,b,c^	60 (67.4)^b,c^
S_10_	113	54 (48.01)^c^	6.77 ± 0.4	11.17 ± 1.0	13 (11.7)^c^	57 (50.4)^b^	17 (15.0)^c,d^	117	22 (19.6)^b,c^	74 (62.7)^c^
S_12_	128	76 (59.1)^b^	6.87 ± 0.3	11.5 ± 0.7	12 (9.6)^c,d^	52 (40.9)^c^	15 (11.7)^d,e^	102	15 (14.7)^c,d^	61 (59.5)^c,d^
S_14_	131	98 (74.0)^a^	6.88 ± 0.4	11.7 ± 0.6	9 (6.9)^d^	31 (24.6)^d^	13 (10.0)^e^	113	11 (10.1)^d^	63 (55.8)^d^

^A)^ Pollen mother cells

^B)^ Metaphase I

^C)^ Anaphase I.

We hypothesized that genome instability of Chh could be primarily responsible for the reduced fertility of *C*. *×hytivus*. Nevertheless, the frequency of PMCs with 19 homologous bivalents increased significantly with generations. Correspondingly, the frequency of meiotic abnormalities, including univalents, intergenomic pairing, and lagging chromosomes, decreased significantly (Table 2). In line with this, pollen stainability increased steadily by generation, suggesting the recovery of fertility (Figure 3a).

### 2.4 Broadened Genetic‐Based and ‐Enhanced Heat Resilience

The initial gene prediction identified 72 and 82 NBS‐LRR‐encoding genes in the Chc and Chh subgenomes of *C. ×hytivus*, respectively (Tables S26 and S27, Supporting Information). Of these, 79.87% (64 Chc and 59 Chh) were colinear with those of CC and HH (Figure 2b; Table S27, Supporting Information). The retention of the most duplicated NBS‐LRR encoding genes from both parents could provide *C. ×hytivus* with more resilience to diseases, increasing its chance to survive where the parent species cannot.^[^ ^30^ ^]^ Indeed, *C. ×hytivus* (S_14_) showed resistance to RKN (*Meloidogyne* spp.) comparable to that of HH,^[^ ^31^ ^]^ and higher than that of CC (Figure S11, Supporting Information).

**Figure 5:**
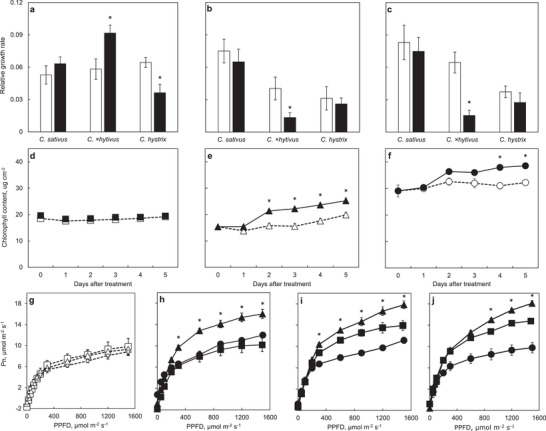
a–c) RGR of *C. ×hytivus* and diploid parents under control (white bars) and high temperature (black bars) for 5 days: a) plant height, b) leaf length, and c) leaf width. d–f) Chl content of developing leaf in the three species measured by Dualex 4 from day 0 to day 5: d) *C. sativus* (CC), e) *C. ×hytivus*, and f) *C. hystrix* (HH). g–j) Light response curves of Pn of the three species g) before the treatments, h) on day 1, i) day 2, and j) day 5 of the treatments: *C. sativus* (CC) (square); *C. ×hytivus* (HHCC) (triangle), and *C. hystrix* (HH) (circular). Control (white dotted line) and HT treatment (black solid line). Vertical bars represent the mean values ± SD (*n* = 3). An ANOVA was performed to test the differences between the control and HT treatment. Mean separations were done using the Duncan multiple range test of *p* < 0.05.

In addition, polyploidy confers resistance to abiotic stresses not tolerated by diploid progenitors.^[^ ^32^ ^]^ We compared the growth, physiological response, and transcriptomic expression levels in the leaves of *C. ×hytivus* compared with its two diploid parental species exposed to elevated temperature and control conditions. The relative growth rates (RGR) of plant height were significantly increased in *C. ×hytivus* under heat treatment for 5 days, while the RGR of leaf size was significantly decreased (**Figure** **5**a–c). The chlorophyll (Chl) content and net photosynthesis rate (Pn) of *C. ×hytivus* significantly increased after 2 days and 1 day of heat treatment, respectively (Figures 5e and 5h). Gene expression analysis showed that the expression of 2135 genes was significantly changed after heat treatment in *C. ×hytivus*, but not in the parents (Figure S12a, Supporting Information). These genes were mainly involved in carbon fixation in photosynthetic organisms, carbon metabolism, and glyoxylate and dicarboxylate metabolism, which confirmed the observed enhanced Chl accumulation and photosynthesis of *C. ×hytivus* in response to heat stress (Figure S12b, Supporting Information).

## 3 Discussion

In this study, we present the first chromosome‐scale genome assembly of a synthesized allotetraploid. Thereafter, by systematic sampling through generations of inbreeding after the initial polyploidization, we were able to precisely identify the nature of genomic changes that emerged through successive rounds of inbreeding, and tested for changes in underlying adaptability to stress. Our results reveal a detailed set of mechanisms that likely account for the phenomenon of subgenome dominance, which has been described in many allopolyploid species,^[^ ^26^ ^]^ including *Arabidopsis thaliana*,^[^ ^7^ ^]^ *Zea mays*,^[^ ^33^ ^]^ *Brassica rapa*,^[^ ^30b^ ^]^ *B. juncea*,^[^ ^19^ ^]^ *Triticum aestivum*,^[^ ^34^ ^]^ and *Arachis hypogaea*.^[^ ^24^ ^]^ Some allopolyploids such as *Capsella bursa‐pastoris* do not exhibit subgenome dominance.^[^ ^35^ ^]^ Pumpkin or squash (*Cucurbita* spp.), belonging to the same Cucurbitaceae family as *Cucumis*, is a paleo‐allotetraploid. No significant dominance was found between the two ancient subgenomes of *Cucurbita*, perhaps due to their similar TE numbers and distributions.^[^ ^8^ ^]^ In a previous study on *C. ×hytivus*, preliminary amplified fragment length polymorphism (AFLP) analysis showed that the frequency of sequence loss from the CC genome was higher than that from the HH genome in both the initial F_1_ hybrid and S_0_ generation.^[^ ^18d^ ^]^ It should be noted, however, that AFLPs are dominant markers; therefore, it is difficult to infer genome‐wide conclusions from them. In contrast to the previous study, in the present work, genome‐wide analysis was carried out via sequencing that demonstrated that the CC‐derived Chc subgenome experienced significantly less fractionation and was more highly expressed than the HH‐derived Chh subgenome following the experimental allopolyploidization that synthesized *C*. *×hytivus*. This bias (“dominance”) begins to manifest immediately after interspecific hybridization, consistent with the results from a recent report on monkeyflowers.^[^ ^9^ ^]^

Allopolyploidization involves two processes: genome merger (e.g., hybridization of different genotypes, typically from different species) and genome duplication. Synthetic allopolyploid systems allow direct comparison of the unduplicated F_1_ hybrid with its doubled offspring, and thus represent an important tool for understanding the early stages of allopolyploid formation,^[^ ^5^, ^14^ ^]^ and for assessing the relative contributions of genome merger and genome doubling.^[^ ^14^ ^]^ Given the importance and prevalence of allopolyploidy in plants,^[^ ^36^ ^]^ relatively few studies have been conducted to date, and even among these, results among distinct genera differ.^[^ ^37^ ^]^ In *Arabidopsis* allotetraploids, changes in gene expression were primarily attributed to interspecific hybridization rather than polyploidization.^[^ ^38^ ^]^ Similarly, hybridization rather than genome doubling is reported to trigger the majority of genetic and epigenetic changes in *Spartina*.^[^ ^39^ ^]^ In contrast, although most sequence elimination was attributed to hybridization in one cross between two species of wheat (*Aegilops sharonensis* × *A. umbellulata*), it was a chromosome duplication that led to more sequence loss in another cross of two wheat species (*A. longissima* × *T. urartu*).^[^ ^40^ ^]^ Furthermore, a study of a *Senecio* allohexaploid suggested that the two events could have distinct effects on gene expression: changes in gene expression induced by hybridization may have been ameliorated by genome duplication.^[^ ^41^ ^]^ Our results suggest that genome duplication may also have a recovery effect on genome structure, and many missing genes in the F_1_ diploid hybrid reappeared in the duplicated allotetraploid (Figure 3b). It also supports our earlier AFLP analysis that some of the parental fragments lost at the hybrid stage reappeared after allopolyploid formation.^[^ ^18d^ ^]^ However, the underlying mechanism of regaining the lost gene needs to be further uncovered. Although bioinformatic inference of gene loss is widely applied in polyploid studies, it should also be noted that statistical false results, for example, false read alignment, are also possible, since the definition of gene loss is based on the artificially calculated threshold value.^[^ ^19^, ^42^ ^]^ Nevertheless, all these observations lead to the conclusion that the effects of interspecific hybridization and genome duplication on shaping the genome of allopolyploids are species dependent, and it is more common that hybridization has a larger impact than duplication, as is the case in our *Cucumis* allotetraploid. More genome‐wide analysis taking advantage of sequencing technology of different synthetic allopolyploids and their F_1_ progenitors in the future would help to discover which of the two processes is generally more important in the genomic shock phenomenon.

Changes occurred not only during the process of allopolyploidization, but also in subsequent generations (post‐allopolyploidy). Rapid genomic reshaping is frequently observed in many synthetic allopolyploids, such as wheat (*T. aestivum*),^[^ ^43^ ^]^ *Brassica*,^[^ ^44^ ^]^ and *Tragopogon*.^[^ ^45^ ^]^ Analyses of the *Aegilops*–*Triticum* complex (wheat and its relatives) revealed that changes in DNA sequence accumulated throughout the first three generations after allopolyploidization.^[^ ^46^ ^]^ In contrast to these observations, no rapid genomic changes were observed after polyploidization in cotton.^[^ ^47^ ^]^ Our previous study using AFLP markers indicated that sequence elimination occurred in the first few generations after polyploid formation, and then slowed down during diploidization.^[^ ^18d,e^ ^]^ These findings were further validated by the present study, which showed that genome‐wide changes happened quickly in the first few generations after allopolyploidization, with fewer changes afterwards. In nature, this diploidization process could last for millions of years, which is still considered “rapid” in plant evolution, for an interspecific hybridization event followed by genome duplication to return to disomic inheritance and become established as a new species.^[^ ^48^ ^]^ According to estimates, the percentage of stained pollen of *C. ×hytivus* recovered to over 80% after 14 more generations. Compared to the natural evolutionary time scale, our results showed that relatively stable (recovered fertility and diploid‐like meiotic behavior) allopolyploids could be obtained relatively rapidly through diploidization following artificial polyploidization, which is promising for crop improvement via polyploidy.

Given the widespread distribution and evolutionary and ecological success of allopolyploid species, it has been inferred that this genomic structure may be advantageous, owing to various attributes, notably fixed heterozygosity.^[^ ^49^ ^]^ The retention and persistence of duplicate versions of expressed genes in allopolyploids may facilitate genetic robustness and adaptation to environmental changes.^[^ ^38a^ ^]^ For instance, most of the resistance genes from both parents were retained in *C. ×hytivus*, including those genes from HH that were absent in CC. Increased disease resistance of *C. ×hytivus* relative to CC indicates the feasibility and potential utility of transferring useful resistance genes from wild relative species to cultivated species by artificial polyploidization. Enhanced abiotic stress tolerance has been observed in some allopolyploids.^[^ ^32^, ^50^ ^]^ Allopolyploids may reach a new transcriptional homeostasis under stress by regulating duplicate gene expression to accelerate phenotypic adaptation,^[^ ^51^ ^]^ as is indicated by our results. Studies on hexaploid wheat suggested that condition‐dependent functionalization of the duplicated genes from subgenomes might have contributed to the improved adaptability.^[^ ^52^ ^]^ In addition, our SV analysis showed that polyploidization‐induced SV was involved with a various of biological processes, including plant hormone signal transduction, plant–pathogen interaction, and photosynthesis (Table S28, Supporting Information), suggesting that de novo mutations accumulated after polyploidization may also contribute to its wide‐ranging adaptability. Therefore, the factors that cause the increased tolerance of polyploids could vary, such as de novo mutations, transcriptomic regulation of duplicated genes, new gene interactions, and so on. Studies on different polyploid experimental systems may also result in various outputs. More evidence is needed to fully reveal the underlying mechanism of polyploidization‐driven tolerance to harsh environmental conditions. Nevertheless, considering predicted global warming and rising frequency of extreme climate events, possible advantageous adaptability resulting from artificial allopolyploidization has implications for developing tolerant species/varieties to feed the world's growing population in a challenging climate.

## 4 Conclusion

In this study, we report the high‐quality genome of a synthetic allotetraploid obtained using interspecific hybridization between cucumber (*C. sativus*) and its wild relative species (*C. hystrix*) and subsequent chromosome duplication, which is the first fully sequenced synthetic allopolyploid. By precise comparative analysis with parental genomes, we demonstrated the dominance of the *C. sativus*‐originated subgenome, although both subgenomes largely maintained the chromosome structure of their diploid parents. We also sequenced the genomes of the F_1_ homoploid hybrid, the original duplicated allotetraploid (S_0_), and the subsequent generation individuals (S_4_–S_13_). Our results indicate that hybridization, rather than genome duplication, causes the majority of genomic changes in both nuclear and cp genomes. Moreover, post‐polyploidy genomic changes occurred mainly in the first few generations and slowed down afterwards. By testing the RKN resistance and heat tolerance, we suggested that the fixed heterozygosity provides *C*. ×*hytivus* with increased stress adaptation. Our results provide new insights into plant polyploidy evolution and offer a prospective breeding strategy for future crops.

## 5 Experimental Section

##### Plant Materials

Inbred lines of the two diploid parents (*C. sativus* L. var “Beijingjietou” and *C. hystrix* Chakr.), their interspecific F_1_ homoploid hybrid, and synthetic allotetraploid *C. ×hytivus* were used. Different generations (S_4_–S_14_) of *C. ×hytivus* were obtained by continuing self‐pollination with the original duplicated F_1_, named S_0_. In each generation, individual self‐pollination was performed, seeds obtained were mixed, and several seeds were randomly chosen and planted to generate the next generation. The original F_1_ homoploid hybrid and S_0_ were preserved via tissue culture. The highly inbred synthetic allotetraploid, *C. ×hytivus* (S_14_), was chosen as the reference for genome sequencing using SMRT sequencing technology (Pacific Biosciences). The original F_1_ homoploid hybrid, S_0_, and one individual from each generation (S_4_–S_13_) of *C*. *×hytivus* were also used for genome sequencing using Illumina short‐read technology for comparative genomics analysis. Six generation plants of *C. ×hytivus* (S_4_, S_6_, S_8_, S_10_, S_12_, and S_14_) were chosen for meiotic analysis. All the materials were grown in a greenhouse at Baima Teaching and Research Base of Nanjing Agricultural University, Nanjing, China, unless special conditions are mentioned. It should be noted that the individual *C. hystrix* used for genome sequencing is not the one for interspecific hybridization, but its self‐crossed progeny. The male parent *C. sativus* L. var “Beijingjietou” is a close cucumber cultivar to the sequenced cucumber “Chinese long” inbred line 9930.

##### Heat Treatment

Seeds of *C. ×hytivus* (S_14_) and inbred lines of diploid parents, *C. sativus* L. var. “BeijingJietou” and *C. hystrix* Chakr., were sown in plastic pots of 11 × 11 × 6 cm (length × width × height) filled with peat‐based potting mix (Pindstrup 2, Pindstrup Mosebrug A/S, Ryomgaard, Denmark). Uniform seedlings with three true leaves were transferred to controlled climate chambers. Temperature treatment was conducted in these controlled climate chambers with heat treatment defined as (38 °C/30 °C day/night) contrasted with control conditions that were not heat stressed (28 °C/20 °C day/night), respectively. The photoperiod was set to 14/10 h day/night, light intensity was 500 µmol m^−2^ s^−1^, and air humidity (AH) was ≈75%. Irrigation was done every morning by flooding the seedling for 10 min with nutrient solution containing N (196 mg L^−1^), P (31 mg L^−1^), K (234 mg L^−1^), and Mg (43.2 mg L^−1^) along with micronutrients.

##### Physiological Measurements and Data Analysis

RGR was calculated from plant height, stem diameter, leaf length, and leaf width from the control and HT treatments using the formula: RGR = [ln (M_2_) − ln (M_1_)]/(T_2 _− T_1_) (M_1_: measurement 1, M_2_: measurement 2, T_1_: measurement time 1, and T_2_: measurement time 2).

During the experiment, the change in Chl content was noninvasively monitored via Dualex 4 (FORCE‐A, Orsay, France).^[^ ^53^ ^]^ Dualex 4 measures Chl contents in micrograms per square centimeter. The mean value of each leaf was calculated from three sections on both sides. On day 0, the first unfolded leaf was measured, and the same leaf was used throughout the treatment.

Pn, stomatal conductance (Gs), internal CO_2_ concentration (Ci), and transpiration rate (Tr) were measured using a Li‐6400 portable photosynthesis assay apparatus (LI‐COR Biosciences, Inc., USA). The light response curves were measured using a 6400‐02B red and blue light source of the LI‐6400 photosynthesis system. The leaf photosynthetically active radiation (PAR) was controlled at 12 levels from 0 to 1500 µmol m^−2^ s^−1^ (0, 25, 50, 75, 100, 150, 200, 300, 600, 900, 1200, and 1500). During the measurement, the ambient leaf temperature, humidity, and CO_2_ concentration were controlled to a steady state.

##### Genome Sequencing

Fresh young leaves were collected from a single plant for each sample and immediately frozen in liquid nitrogen for 24 h. Genomic DNA for PacBio and Illumina sequencing was extracted using the CTAB method. The PacBio sequencing library was prepared according to the recommendations of Pacific Biosciences. Genomic DNA was fragmented to ≈20 kb targeted size by g‐TUBE centrifuged at 2000 rpm for 2 min, then treated with end‐repair, adapter ligation, and exonuclease digestion. DNA fragments of ≈20 kb in length were collected via BluePippin electrophoresis (Sage Sciences). DNA libraries were sequenced on the PacBio Sequel platform (Pacific Biosciences) using P6‐C4 chemistry. A total of 53.92 G raw data were obtained.

For genome sequencing of 12 samples including F_1_ and early generations (S_0_, S_4_–S_13_), two mate‐pairs (3 and 4 kb), and one paired‐end (270 bp) Illumina libraries were constructed and sequenced according to the standard protocol of the Illumina X‐TEN platform (Illumina, San Diego, CA, USA) for each sample.

##### BioNano Sequencing

Cuttings from the *C. ×hytivus* (S_14_) plant used for genome sequencing were planted in plastic pots (11 cm diameter, 0.5 L) filled with a peat‐based potting mix (Pindstrup 2, Pindstrup Mosebrug A/S, Ryomgaard, Denmark) and irrigated and fertilized regularly with a nutrient solution with N/P/K of 160:35:190, pH 5.8, and electric conductivity of 1.8. They were cultivated in a growth chamber at Nanjing Agricultural University. Young leaves were treated in the dark for 2 days before sampling. High‐molecular‐weight DNA was isolated and labeled with the single‐stranded nicking endonuclease Nt. BssSI following standard BioNano protocols. The labeled DNA sample was subsequently loaded onto the IrysChip nanochannel array, and the stretched DNA molecules were imaged with the BioNano Irys system. Basic labeling and DNA length information were retrieved from bnx files converted from raw image data using AutoDetect software.^[^ ^54^ ^]^

##### Hi‐C Sequencing

Hi‐C libraries were prepared from leaves as described previously.^[^ ^55^ ^]^ Briefly, nuclear DNA was fixed with formaldehyde and then digested with Hind III. Sticky ends were biotinylated, and then diluted and ligated. Biotinylated DNA was enriched and then sheared to ≈350 bp fragment size. The Hi‐C fragment library was constructed and sequenced using the Illumina X‐TEN platform (Illumina, San Diego, CA, USA, 2 × 150 bp) for pseudomolecules construction.

##### RNA‐Seq and PacBio Iso‐Seq

On the sixth day of the chamber heat treatments, the same leaves that were used for physiological measurements were sampled for RNA extraction. Samples were stored in liquid nitrogen until RNA extraction. After RNA extraction, the purity, concentration, and integrity of RNA were tested using Nanodrop, Qubit 2.0, and Agilent 2100, respectively. mRNA‐Seq library construction was performed after obtaining quality samples that were subjected to high‐throughput sequencing using Illumina HiSeq.

For Iso‐Seq, high‐quality RNA was extracted from eight tissues of *C. ×hytivus* (S_14_), including root, stem, leaf, seed, female, and male flowers and fruits (2 and 6 days post‐anthesis), and reverse transcribed. The cDNA was normalized using the Evrogen‐Trimmer‐2 Kit (Evrogen, Moscow, Russia, catalog no. NK003). Tissue‐specific barcodes were added before pooling for subsequent amplification. To avoid loading bias, which favors sequencing of shorter transcripts, multiple size‐fractionated libraries (≈0.5 and ≈2 kb) were constructed using a SageELF device.

##### Genome In Situ Hybridization and Oligo‐Fluorescent In Situ Hybridization

Root tips and young male flower buds of plant materials were collected and fixed in Carnoy's solution at 4 °C for 1 day. The young male flower buds of six different generations were randomly collected and divided into three groups for meiotic behavior analysis. The procedure to prepare samples for analysis was performed as described previously, with some modifications as follows.^[^ ^56^ ^]^ The fixed root tips were digested with an enzyme mixture containing 4% cellulose R‐10 (Yakult), 2% pectinase (Sigma‐Aldrich), and 0.1% pectolase (Yakult) in 0.01 m citrate buffer (pH = 4.8), at 37 °C for 40–60 min. The anthers were collected and digested using enzyme mixtures, including 4% cellulose R‐10 (Yakult), 4% pectinase (Sigma‐Aldrich), and 2% pectolase (Yakult) at 37 °C for 50–70 min (meiotic pachytene) and 2–3 h (meiotic metaphase and anaphase). Finally, the digested root tips and anthers were smeared onto slides. The slides that showed adequately spread chromosomes were prepared for FISH and GISH experiments.

An oligo library developed from *C. sativus* chromosome C04 was designed using Chorus software (https://github.com/forrestzhang/Chorus). The repeat sequences in cucumber genome sequences were filtered using RepeatMasker (http://www.repeatmasker.org/). The oligos (50 nt) specific to “the Chinese long cucumber” genome (http://cucurbitgenomics.org/organism/20, v3 Genome) were selected throughout filtered sequences of chromosome C04 with a step size of 25 nt. The oligos located at CDS and single‐copy regions were selected preferentially to ensure utility for cross‐species FISH painting. A total of 93 396 oligos were generated for cucumber chromosome C04. As previously described, the oligo library was divided into eight sub‐pools to perform chromosomal segmentation painting to illustrate the syntenic relationship of chromosomes involved, and synthesized by Synbio Technologies (Suzhou, China, http://www.synbio-tech.com.cn). The oligo probes were synthesized using a published protocol as follows.^[^ ^26^ ^]^ Briefly, 50 µL of PCR mixture consisted of ≈0.14 ng DNA from the oligo library, 2 µL of 1 × 10^−3^ m fluorophore‐tagged F primer, 2 µL of 1 × 10^−3^ m fluorophore‐tagged R primer, 25 µL of HiFi HotStart ReadyMix (KAPA, Kit Code, KK2601), and 18 µL of nuclease‐free water. The PCR mixture was incubated at 95 °C for 3 min, followed by 15 cycles of 98 °C for 20 s, 60 °C for 30 s, 72 °C for 30 s, then 25 cycles of 98 °C for 20 s, 55 °C for 30 s, 72 °C for 30 s, with a final extension at 72 °C for 1 min. The PCR reaction was cleaned with the GeneJET PCR Purification kit (Thermo Fisher Scientific, Kit Code, K0702) and eluted with 40 µL solution buffer to obtain labeled oligo probes for chromosome painting.

FISH was performed essentially as described previously.^[^ ^57^ ^]^ The hybridization mixture containing 10 µL of 100% formamide, 2 µL of 20× SSC, 4 µL of 50% dextran sulfate, and 3 µL oligo probes (>500 ng) was denatured at 90 °C for 6 min, then transferred to ice and incubated for at least 5 min. The hybridization mixture was then applied to denatured chromosome slides and incubated overnight at 37 °C. Slides were washed for 5 min in 2× SSC at room temperature (RT), then for 10 min in 2× SSC at 42 °C, and then for 5 min in 2× SSC at RT for 5 min in 1× PBS at RT. The washed slides were air‐dried in the dark, and then counterstained with DAPI in VECTASHIELD Antifade solution (Vector Laboratories).

For GISH treatments, genomic DNA was extracted from cucumber and *C. hystrix* using the CTAB method, and then labeled as GISH probes for distinguishing the two subgenomes of allotetraploid *C. ×hytivus* during meiosis.^[^ ^57^ ^]^ All experimental procedures for GISH were performed as previously described.^[^ ^56^ ^]^ FISH and GISH images were captured using a SENSYS (http://www.photometrics.com) CCD camera attached to an Olympus (http://www.olympus-global.com) BX51 microscope. The CCD camera was controlled using FISH view 5.5 software (Applied Spectral Imaging, Inc., http://www.spectral-imaging.com). Images were processed using Adobe Photoshop CC (Adobe Systems, http://www.adobe.com). Pachytene chromosomes were straightened using ImageJ software (https://imagej.nih.gov/ij/).

##### Chloroplast DNA Isolation and Sequencing

About 10 g fresh leaves were sampled from adult plants of *C. hystrix*. An improved sucrose gradient centrifugation method was used to isolate the total cp DNA.^[^ ^58^ ^]^ The quality of genomic DNA was checked by monitoring A260/A280 ratios (DU800, Beckman Coulter, USA) and Tris‐borate‐EDTA polyacrylamide gel electrophoresis. DNA was randomly fragmented by sonication. The resulting fragments were subsequently subjected to end‐repair and phosphorylation using T4 DNA polymerase, Klenow DNA polymerase, and T4 Polynucleotide Kinase. Thereafter, an “A” base was inserted as an overhang at the 3ʹ ends of the repaired DNA fragments and Illumina paired‐end adaptors were subsequently ligated to these DNA fragments to distinguish the different sequencing samples. Finally, the library was sequenced using an Illumina HiSeq 2000 instrument according to the manufacturer's instructions (Illumina, San Diego, CA).

##### RKN (*Meloidogyne* spp.) Resistance Determination

Before germination, seeds of *C. sativus*, *C. hystrix*, and *C. ×hytivus* (S_14_) were surface sterilized with 5% sodium hypochlorite solution for 10 min, and rinsed with distilled water three times. Sterilized seeds were placed on wet filter paper in Petri dishes, and incubated in a growth chamber at 28 °C. Seedlings were then sown individually into 11 × 11 × 6 cm (length × width × height) plastic pots filled with steam‐sterilized sand. Air temperatures in the greenhouse were maintained at ≈30 °C during the day and 24 °C at night. A randomized complete block experimental design with four biological replications was used. Two‐week‐old plantlets were inoculated with ≈400 second‐stage juveniles (J2 s) of *Meloidogyne incognita* race 1 at the root tip using a pipette tip. Thirty days after inoculation, each plant was uprooted, the roots were washed free of soil, and the *M. incognita* galls were counted. The reproduction rate of *M. incognita* was determined according to a published protocol.^[^ ^59^ ^]^

##### Data Availability

Raw genome sequence reads of *C. ×hytivus* have been deposited at DDBJ/ENA/GenBank under the accessions number PRJNA594754. The genomic data of *C. hystrix* are available at Figshare (https://doi.org/10.6084/m9.figshare.13377671). The raw PacBio Iso‐Seq reads and transcriptome sequence reads were deposited at the NCBI sequence read archive under accessions SRP262554 and SRP155470, respectively. The clean Illumina sequencing reads of actual male parent, *C. sativus* L. var. “Beijingjietou,” was deposited at the NCBI sequence read archive under accession SRP284803. The genome sequences and annotations of *C. sativus*, *C. hystrix*, and *C. ×hytivus* are also available in the *Cucumis* Genome Database (http://www.cucumisgdb.cn/). All materials and other data in this study are available upon reasonable request.

##### Statistical Analysis—Physiological Data Analysis

For Chl content measurement, three random plants from each species were selected from each treatment and measured (*n* = 3), and the standard deviations (SD) were considered as the error line. A two‐way analysis of variance (ANOVA) was performed to test the differences between the control and HT treatment over a 5 day period of measurements. The software R (i3862.15.0, www.r-project.org/) was used for statistical analysis. Mean separations were performed using Duncan's multiple range test of *p* < 0.05.

##### Genome Assembly

The raw polymerase reads were processed using the PacBio SMRT‐Analysis package (https://www.pacb.com/products-and-services/analytical-software/smrt-analysis/) to remove sequencing adapters and filter low quality and short length reads (parameters: readScore, 0.75; minSubReadLength, 500). Considering the high error rate of PacBio reads, an error correction module embedded in Canu (correctedErrorRate: 0.045) was first used to correct the reads.^[^ ^60^ ^]^ Next, the resulting high‐quality PacBio sub‐reads were used for genome assembly with Canu software (Table S29, Supporting Information).^[^ ^60^ ^]^ The assembled contigs were supported by mapping 96.79% of clean sub‐reads (with sequence length >10 kb) for *C. ×hytivus* (S_14_) using BLASR.^[^ ^61^ ^]^ Finally, consensus sequences of assembly were subjected to mapping of ≈50‐fold coverage of Illumina pair‐end reads using BWA^[^ ^62^ ^]^ and were polished using Pilon software (parameters: –mindepth 10 –changes –fix bases). An independent whole‐genome sequence assembly was executed using SOAPdenovo2 packages for each sample of the F_1_ and early generations (S_0_, S_4_–S_13_). From over 80‐fold coverage reads (≈60 Gb), 469–491 Mb results were assembled with scaffold N50 and contig N50 of 134–226 kb and 47–73 kb, respectively (Table S30, Supporting Information). To decrease the chimeric sequences in initial assembly results, different fragment mate‐paired data were mapped to the contigs using BWA,^[^ ^62^ ^]^ considering only unique mapping reads for further scaffold construction. Scaffolding was performed via SSPACE^[^ ^63^ ^]^ using two mate‐pair data and estimating gaps between the contigs according to the distance of MP links. Two contigs supported by at least five reasonable MP links in each fragment library (insert size ±5 SD) were joined as a scaffold.

##### Scaffolding Using Optical Maps of the BioNano System

In total, 163.4 Gb single molecule data for *C. ×hytivus* (S_14_) were obtained after filtration by a molecule length ≥150 kb with a signal‐to‐noise ratio (SNR) ≥3.0, average molecule intensity <0.6, and labels ≥8 per molecule. High‐quality labeled molecules were pairwise aligned, clustered, and de novo assembled into a consensus map following the Assembler software developed by BioNano Genomics (http://www.bionanogenomics.com/). A physical map was assembled with a total length of 499.04 Mb (Table S1, Supporting Information). The in silico map from contigs assembled from PacBio subreads was aligned with the optical consensus map using RefAligner. Anomalies in the PacBio‐based assembly and consensus map were corrected, and then PacBio‐based contigs were extended using Irys‐scaffolding with default parameters. The hybrid assembly was obtained with a length of 540.74 Mb and scaffold N50 8.09 Mb. Thereafter, the clean Illumina short reads were mapped back to the assembly for SNP calling and 1067 homozygous SNPs were obtained, and the single base error rate was 0.0001973192%.

##### Chromosome‐Scale Assembly Using Hi‐C

Raw Hi‐C data were processed to filter low‐quality reads, and adapters were trimmed with cutadapt (RRID: SCR 011841).^[^ ^64^ ^]^ The clean Hi‐C reads were then mapped to the assembly results genome of *C. ×hytivus* (S_14_) with BWA (mapping method: aln).^[^ ^62^ ^]^ Only unique mapped read pairs (58.13%) were considered for further analysis (Table S4, Supporting Information). Duplicate removal, sorting, and quality assessment were carried out using HiC‐Pro.^[^ ^65^ ^]^ Of the Hi‐C data, 59.00% were valid interaction pairs. Next, the uniquely mapped data were retained for assembly using LACHESIS.^[^ ^66^ ^]^ Hi‐C data were used to correct mis‐joins in contigs, and then to order and orient contigs. Pre‐assembly was performed for contig correction by splitting contigs into segments with an average length of 50 kb, and then the segments were pre‐assembled with Hi‐C data. Misassembled points were identified and broken at the likely point of misassembly when split segments could not be placed in the original position. Next, the corrected contigs were assembled using LACHESIS with parameters CLUSTER_MIN_RE_SITES = 225, CLUSTER_MAX_LINK_DENSITY = 2; ORDER_MIN_N_RES_IN_TRUN = 105; ORDER_MIN_N_RES_IN_SHREDS = 105 with Hi‐C valid pairs. Gaps between ordered contigs were filled with 100 N's. Based on 104‐fold coverage of Hi‐C data, the vast majority (97.23%) of the assembled sequence was anchored onto the 19 pseudo‐chromosomes via frequency distribution of valid interaction pairs (Table S5, Supporting Information). To assess the quality of assembly, Hi‐C data were mapped to chromosomes using HiC‐Pro.^[^ ^65^ ^]^ The interaction matrix was visualized with a heatmap at the 100 kb resolution (Figure S4, Supporting Information).

##### RNA‐Seq and Iso‐Seq

Three biological replicates were used for RNA‐seq. Empty reads, adapter sequences and low‐quality sequences were removed from raw reads to obtain clean reads. A total of 135.86 Gb clean data were obtained. The clean data of each sample reached 6.10 Gb and a Q30 base percentage of 91.37% or higher. For Iso‐Seq, although PacBio single molecule sequencing yields long reads, it has a high error rate. Using the Iso‐Seq protocol, the error rate is lower because multiple subreads in the same zero‐mode waveguides produce a read of insert (ROI) (also known as circular consensus sequence) with higher accuracy. Consequently, 329 978 ROIs were obtained, of which 203 354 were full‐length ROIs (containing 5ʹ primer, 3ʹ primer, and poly (A) tail). The rest were non‐full‐length ROIs (Table S7, Supporting Information).

##### Repeat Analyses

Tandem repeat composition analysis was performed according to previous method.^[^ ^67^ ^]^ All Illumina reads of *C. ×hytivus* (S_14_) were aligned against each type of tandem repeat using BLASTN with an e‐value of 1e‐10. The reads were considered as repeat if the length of alignment was over 100 bp or the coverage of the read was over 70%. Type I/II, III, and IV repeats in cucumber were retrieved from GenBank. Two rDNA (45S and 5S) sequences were obtained previously.^[^ ^68^ ^]^

The repeat sequences of *C. ×hytivus* (S_14_) and the parent species (*C. hystrix* and *C. sativus* L. var. 9930) were distinguished using a combination of de novo and homolog strategies. Four de novo programs, including RepeatScout,^[^ ^69^ ^]^ LTR‐FINDER,^[^ ^70^ ^]^ MITE‐Hunter,^[^ ^71^ ^]^ and PILER,^[^ ^72^ ^]^ were used to construct the initial repeat library. The initial repeat database was classified using PASTEClassifier,^[^ ^73^ ^]^ and three *de novo* libraries from *C. sativus*, *C. hystrix*, and *C*. ×*hytivus* were then merged with the known Repbase database.^[^ ^74^ ^]^ Finally, the merged repeat database was used to distinguish the genome assembly repeat sequences using RepeatMasker (Table S6, Supporting Information).^[^ ^75^ ^]^

##### Gene Annotation

Genes were annotated using a combined strategy of three approaches: de novo, homology‐based, and transcript‐based. These results were finally merged with evidence modeler (EVM) (Figure S6 and Table S8, Supporting Information).^[^ ^76^ ^]^ For de novo prediction, Genscan,^[^ ^77^ ^]^ Augustus,^[^ ^78^ ^]^ GlimmerHMM,^[^ ^79^ ^]^ GeneID,^[^ ^80^ ^]^ and SNAP^[^ ^81^ ^]^ were used to scan the repeat‐masked genome. The protein sequences from five sequenced eudicot species, including *A. thaliana* (TAIR10), *Oryza sativa* (MUSv7.0), *Citrullus lanatus* (watermelon (97103) genome v2), *C. melo* (melon (DHL92) genome 3.5.1) and *C. sativus* (cucumber (Chinese Long) genome v3), were used for homology‐based prediction through GeMoMa.^[^ ^82^ ^]^ In the third approach, the Hisat^[^ ^83^ ^]^ and Stringtie^[^ ^84^ ^]^ programs were used to carry out reference‐based transcriptome assembly. GeneMarkS‐T^[^ ^85^ ^]^ was used to predict genes based on transcripts. PASA software was used to predict genes based on unigenes and full‐length transcripts from PacBio sequencing.^[^ ^86^ ^]^ The gene annotation result was evaluated by identifying 448 (97.82%) conserved eukaryotic genes and 1309 (90.90%) complete BUSCO hits (Table S11, Supporting Information). All the predicted genes were annotated by searching the GenBank Non‐Redundant (NR, 20150226), TrEMBL (20151014), Pfam (30.0), Swiss‐Prot (20151014), eukaryotic orthologous groups (KOG, 20110125), GO (20160907), and KEGG (20170310) databases (Table S10, Supporting Information).

##### Pseudogene Prediction and Non‐Coding RNA Annotation

The whole genome was scanned with GenBlastA after masking predicted functional genes.^[^ ^87^ ^]^ Pseudogenes were confirmed by searching for internal stop codons and frame‐shift mutations using GeneWise.^[^ ^88^ ^]^ Non‐coding RNAs (ncRNAs) were predicted using the software Infernal^[^ ^89^ ^]^ based on the Rfam database and miRBase database for rRNA and microRNA, respectively. The tRNAscan‐SE program was applied to detect reliable tRNA positions.^[^ ^90^ ^]^ Summary of non‐coding RNAs and pseudogenes are presented in Table S12, Supporting Information.

##### Syntenic Orthologous Gene Pair Identification and Gene Loss Analyses

The previous assemblies of the *C. hystrix* genome (https://doi.org/10.6084/m9.figshare.13377671) and *C. sativus* L. var. 9930 (v3) were used for comparative analysis.^[^ ^20^ ^]^ Syntenic orthologous gene pairs and syntenic blocks were identified using the QUOTA‐ALIGN package.^[^ ^91^ ^]^ The two diploid parents (*C. sativus* and *C. hystrix*) were mapped to the corresponding subgenomes (Chc and Chh) of *C. ×hytivus* (S_14_), to allow calling of syntenic blocks. First, all‐against‐all BLASP^[^ ^92^ ^]^ alignment was performed with parameters ‐v = 5 ‐b = 5 ‐e = 1e‐5 between *C. sativus* and Chc subgenome and then chained the BLASP hits using QUOTA‐ALIGN (cscore = 0.9)^[^ ^91^ ^]^ with “1:1 synteny screen.” The distance cut‐off of 20 genes was adopted for syntenic block identification. At least four gene pairs were required for individual synteny blocks. Similarly, four pairwise comparisons were performed including Chc to CC (orthologs), Chc to HH (orthologs), HH to CC (orthologs), and Chc to Chh (homeologs) to generate the syntenic relationship and syntenic homologous gene pairs set between two subgenomes (Tables S14 and S15, Supporting Information).

For genes within syntenic blocks, potential gene loss in the Chc subgenome was defined using the following metrics: 1) gene located in CC‐Chc synteny block and 2) gene present in syntenic block of its ancestral genome CC but unable to find homologs within five syntenic gene pairs of corresponding Chc syntenic blocks. These candidate genes missing from the derived subgenome were then considered “potential lost genes.” These genes were first checked in unanchored scaffolds or contigs to exclude false gene loss due to the assembly. Further, to avoid false positives in calling genes because of misassembly and/or mis‐annotation, the protein of the potential lost gene was further mapped in CC to the corresponding syntenic DNA sequences in Chc and identified the potentially miss‐annotated gene (Table S16, Supporting Information). GeMoMa^[^ ^93^ ^]^ was used to identify the miss‐annotated gene using a homolog‐based strategy in Chc. The coding sequence of newly predicted genes from the results of GeMoMa packages in Chc were aligned to the reference genes of “potential lost genes” in CC using BLASTP. If the newly predicted gene was homologous to the reference gene at an identity ≥95% with coverage (alignment length/query, subject) ≥90% and the gene locus was within five adjacent syntenic gene pairs, a candidate lost gene was considered a false positive. In the second step, high‐confidence partially lost genes were removed. If the candidate genes lacked a start or stop codon, they were defined as “partial loss.” If the candidate gene had pseudogenes with frameshift mutations in the homologous region of Chc, they were defined as “pseudogenes.” GeneWise^[^ ^88^ ^]^ was used to predict pseudogenes. After the above filtering, the remaining “potential lost genes” were considered to be “DNA loss” and further validated using Illumina short reads generated from the same accession. To validate the lost gene by short reads, ≈50× clean Illumina reads from *C. ×hytivus* (S_14_) were mapped to the artificial tetraploid genome synthesized from the two parents using BWA with parameters ‐k35 ‐O11. For each “DNA loss” gene, the depth was calculated using command “bedtools coverage ‐counts.” Only genes with depth <1× and coverage of gene body <5% were considered as true lost genes.

For genes outside the syntenic blocks, all of them were regarded as “potential gene losses.” These “potential gene losses” were also further validated in the same way as those potential lost genes within the syntenic blocks.

A similar strategy was used to identify gene loss in the other subgenome, Chh. In addition, to eliminate the possible effect of genetic differences between genotypes, the lost genes were further validated from the CC genome by resequencing Illumina reads of *C. sativus* L. var. “Beijingjietou.”

The status of the confirmed deleted genes in *C. ×hytivus* (S_14_) in the unduplicated F_1_ homoploid hybrid and several early generations (S_0_, S_4_–S_13_) of *C. ×hytivus* (Tables S20 and S21, Supporting Information) was further checked by analyzing their Illumina short read coverage as described above. Clean Illumina reads from the F_1_ homoploid hybrid and several early generations (S_0_, S_4_–S_13_) of *C. ×hytivus* were mapped to two parental genomes of *C. sativus* (CC) and *C. hystrix* (HH). All mapping was performed using BWA with parameters ‐k35 ‐O11 to guarantee high‐quality mapping results. The depth and breadth of coverage for each gene were calculated as described above. Genes with a depth of less than onefold coverage and 5% of gene body coverage were inferred to be deletions.

##### Analysis of HE

This study assayed for HE between the Chc and Chh subgenomes in the *C. ×hytivus* (S_14_) by assessing the read depth coverage and sequence identity. The read depth of coverage analysis was performed as follows. The average depth for the whole genome was ≈50×. Regions with double read coverage (75–150×) were considered duplicated, and those with low or no coverage (0–25×) indicated deletions. The high‐quality PacBio sub‐reads of *C. ×hytivus* (S_14_) were mapped to parental *C. hystrix* and *C. sativus* genomes using BLASR with parameters ‐bestn 1 ‐nCandidates 10 ‐minPctIdentity 70 to guarantee that each subread would uniquely align to the parental genome. The average depth was calculated on 5 kb windows. Adjacent duplicated windows with depths greater than the threshold and within ten distant windows were linked together, as well as adjacent deleted windows. A double‐depth region was considered a candidate HE when the mapping length was more than 30% of the query window. Identity analysis using BLASTN with default parameters was performed between corresponding duplicated and deleted homologous regions of two ancestral genomes. Sequence identity analysis was carried out as follows. First, syntenic analysis between both parents was performed using MUMmer with parameter –mum.^[^ ^94^ ^]^ Adjacent syntenic blocks with distance less than 20 kb were linked together to generate more continuous collinear blocks. For each candidate HE, BLASTN alignment between corresponding HE regions within syntenic blocks of two parents was performed to assess the sequence identity between genomic homeologous exchange sequences. Integrating the evidence of read coverage results and homolog between parental subgenomes, HE could be detected with confidence (Tables S18 and S19, Supporting Information).

##### Analysis of SV

The corrected PacBio reads of *C. ×hytivus* (S_14_) were aligned to the genomes of the parental species (*C. hystrix* and *C. sativus* L. var. 9930), respectively, using NanoVar for SV (insertion, deletion, inversion, translocation, and transposition) calling.^[^ ^95^ ^]^ Since small insertions and deletions can be detected with SNP and indel calling, only large SVs (>25 bp) were considered. Subsequently, these initial SVs were verified with Illumina reads by mapping the short reads of *C. ×hytivus* (S_14_) to its ancestral genomes and checked the breakpoint around the SV locus, as supported by soft‐clip alignment reads. Notably, different from the donor *C. hystrix* of the HH subgenome, *C. sativus* L. var. 9930 used as an ancestral species is not the direct donor of the CC subgenome in *C. ×hytivus* (S_14_). Therefore, the Illumina reads of the actual parent line *C. sativus* L. var “Beijingjietou” of *C. ×hytivus* (S_14_) was mapped onto the genome of *C. sativus* L. var. 9930 using BWA‐MEM with default settings to exclude false SVs resulting from different genotypes. The SVs supported by reads of the actual parent line *C. sativus* L. var. “Beijingjietou” may result from the different genotypes of *C. sativus* L. var. 9930 and *C. sativus* L. var. “Beijingjietou.” The remaining SVs were considered as true SVs accumulated after allotetraploid events. Genes related to these true SVs were retrieved and enriched for functional annotation.

##### Analysis of Gene Expression

The clean reads filtered from the raw reads were mapped onto *C. hystrix*, *C. sativus*, and *C. ×hytivus* (S_14_) genome sequences using Hisat.^[^ ^83^ ^]^ The fragments per kilobase per million mapped read values of expression genes were calculated using StringTie.^[^ ^84^ ^]^ Differentially expressed genes between the control and high‐temperature treatments in the three species were screened using DESeq.^[^ ^96^ ^]^ The Benjamini–Hochberg method was used for differential expression analysis, with the *p*‐value corrected, and false discovery rate <0.01, fold change >2. Analysis of homeologous gene expression bias was performed within syntenic gene pairs according to published protocols.^[^ ^19^ ^]^ Differentially expressed gene pairs that passed the twofold change threshold were regarded as biased gene pairs, and classified as either C‐dominant or H‐dominant. The homeologous copy that showed relatively higher levels of gene expression for each of the biased gene pairs was considered dominant; thus, the more weakly expressed homeologue was determined to be subordinate with respect to the expression level in the particular treatment. The remaining syntenic gene pairs that showed no dominance relationship between the homeologues in a designated gene pair were classified as neutral gene pairs. The number of C‐dominant gene pairs, H‐dominant gene pairs, and neutral gene pairs are shown in Table S20, Supporting Information.

##### Analysis of Pollen Viability

Five biological replicates of 15 male flowers randomly collected from each generation of allotetraploid *C. ×hytivus* were assayed for pollen stainability. A minimum of 2000 pollen grains per biological replicate were collected, stained with 1% acetocarmine solution, and counted under a stereomicroscope. The percentage of plump, deeply stained pollen grains was calculated to represent the pollen stainability.

##### Chloroplast Assembly and Annotation

Raw data were cleaned in several steps, including removing reads with unknown bases call (N) more than 10%, removing reads with 20 bp of low quality (≤Q20) bases, removing adapter contamination, and removing duplicated reads. The filtered reads were assembled de novo using the SOAPdenovo,^[^ ^97^ ^]^ and the GapCloser^[^ ^98^ ^]^ software was used to close gaps and finally remove the redundant segment sequence to obtain the final assembly results.

Functional annotations were made using several homologous alignment methods; thus, for a particular sequence, multiple alignment results could be obtained. To ensure biological significance, the annotation retained the optimal match result as a comment for the gene. The assembled sequences were compared with the GenBank Non‐Redundant (NR, 20150226), TrEMBL (20151014), Pfam (30.0), Swiss‐Prot (20151014), eukaryotic orthologous groups (KOG, 20110125), GO (20160907), and KEGG (20170310) databases using the Basic Local Alignment Search Tool (BLAST) to obtain functional annotation information for the encoded gene. The genome of the samples was displayed using Circos (http://www.circos.ca/) software for the assembled genomic sequence of the sequenced sample, combined with the predicted results of the coding gene.

##### SNP and Indel Analysis for Chloroplast Genome

Qualified reads were aligned against the *C. hystrix* cp reference genome with BWA.^[^ ^62^ ^]^ Single nucleotide variants and small insertions and deletions (indels, 2–50 bp) were called by the HaplotypeCaller module of GATK3.4.^[^ ^99^ ^]^ The distribution of SNPs and indels in the *C. hystrix* cp genome was visualized using Circos.^[^ ^100^ ^]^

## Conflict of Interest

The authors declare no conflict of interest.

## Supporting information

Supporting Information

Supporting Table

## Data Availability

The data that support the findings of this study are openly available in DDBJ/ENA/GenBank under the accessions number PRJNA594754, SRP262554, SRP155470 and SRP284803, and in figshare at https://doi.org/10.6084/m9.figshare.13377671.
