# Supplementary material for: Whole‐Genome Sequence of Synthesized Allopolyploids in Cucumis Reveals Insights into the Genome Evolution of Allopolyploidization
Source: Adv Sci (Weinh). 2021 Feb 15;8(9):2004222. doi: 10.1002/advs.202004222 (PMC8097326; doi:10.1002/advs.202004222)
Supplement: Supplementary file 1 — Supporting Information [file ADVS-8-2004222-s001.pdf]

© 2021 Wiley-VCH GmbH

ADVANCED SCIENCE
Open Access

Supporting Information

for *Adv. Sci.,* DOI: 10.1002/advs.202004222

Whole Genome Sequence of Synthesized Allopolyploids in *Cucumis* Reveal Insights into the Genome Evolution of Allopolyploidization

*Xiaqing Yu, Panqiao Wang, Ji Li, Qinzheng Zhao, Changmian Ji, Zaobing Zhu, Yufei Zhai, Xiaodong Qin, Junguo Zhou, Haiyan Yu, Xinchao Cheng, Shiro Isshiki, Molly Jahn, Jeff J. Doyle, Carl-Otto Ottosen, Yuling Bai, Qinsheng Cai, Chunyan Cheng, Qunfeng Lou, Sanwen Huang and Jinfeng Chen**

Supporting Information

Whole Genome Sequence of Synthesized Allopolyploids in *Cucumis* Reveal Insights into the Genome Evolution of Allopolyploidization.

*Xiaqing Yu, Panqiao Wang, Ji Li, Qinzheng Zhao, Changmian Ji, Zaobing Zhu, Yufei Zhai, Xiaodong Qin, Junguo Zhou, Haiyan Yu, Xinchao Cheng, Shiro Isshiki, Molly Jahn, Jeff J. Doyle, Carl-Otto Ottosen, Yuling Bai, Qinsheng Cai, Chunyan Cheng, Qunfeng Lou, Sanwen Huang and Jinfeng Chen**

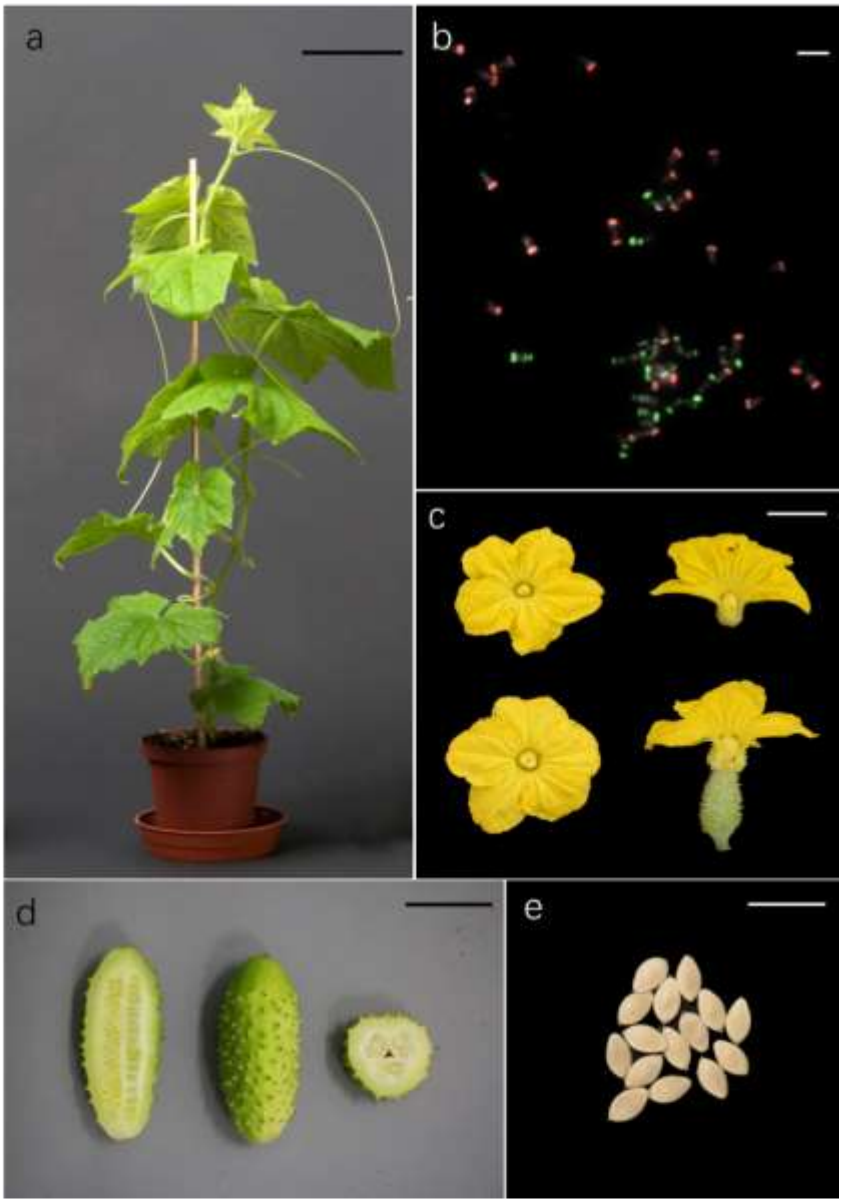

Figure S1. a) Plant, b) mitotic metaphase chromosome pattern, c) flowers, d) fruits, and e) seeds of *C. ×hytivus* (S_{14}). Scale bars indicate a) 10 cm, b) 5µm, c) 1cm, d) 5 cm, and e) 1cm. Red and blue GISH singles in b indicate the Chh and Chc subgenomes respectively.

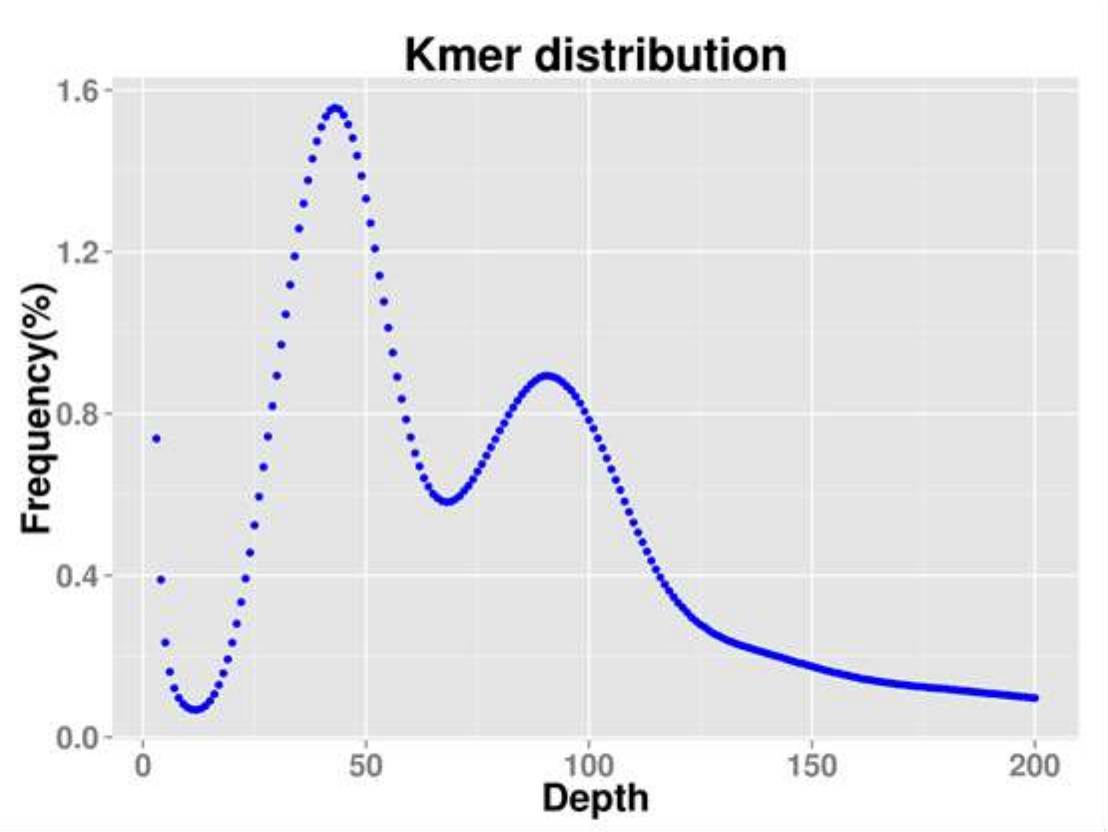

Figure S2. K-mer 17 distribution of *C. ×hytivus* (S_{14}).

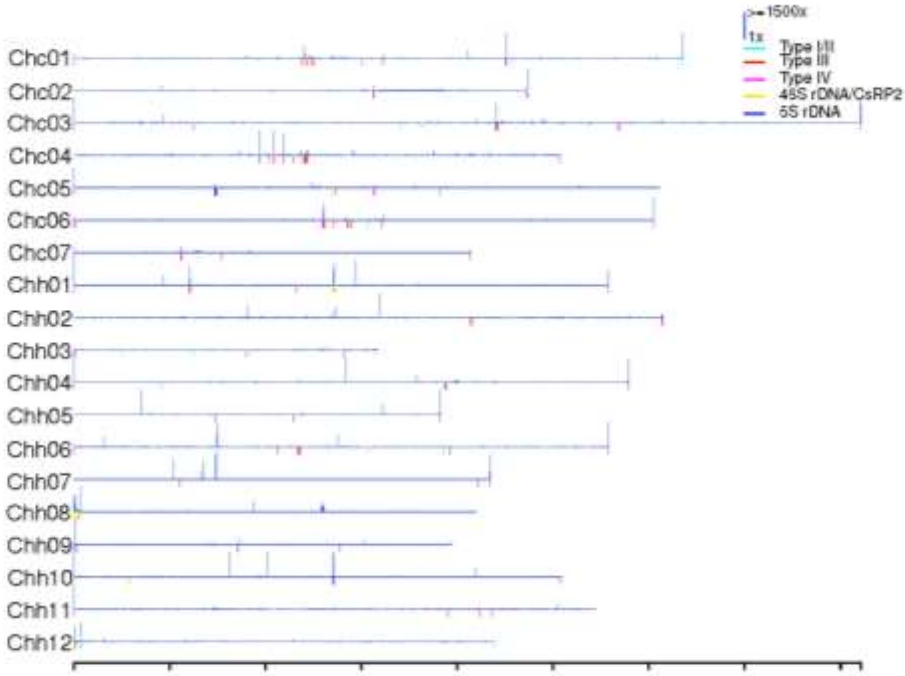

Figure S3. Illumina reads depth on assembly of *C.* ×*hytivus* (S_{14}) in 1-kb sliding windows. Regions that have depth over 1500 × were shown as the highest 1500 ×. The location of each type of repeats were indicated with different color as marked in the top right corner.

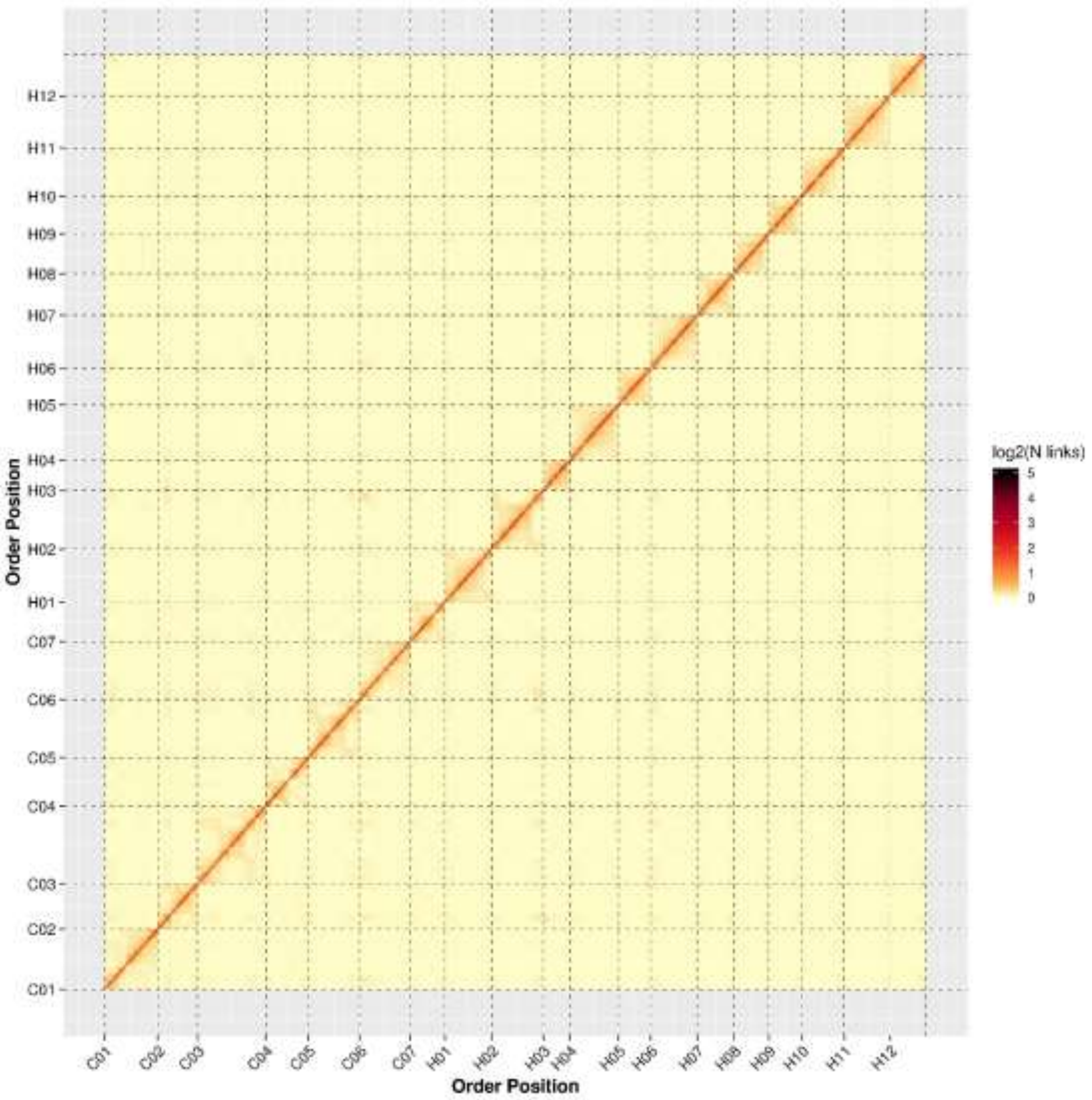

Figure S4. Heatmap of chromosome conformation capture analysis by synthetic intra-chromosomal contact matrix. The intensity of interaction represents the normalized count of Hi-C links between 100 Kb bins on a logarithmic scale. The colored bar on the right side of the figure indicates the strength of interaction, from low (yellow) to high (red).

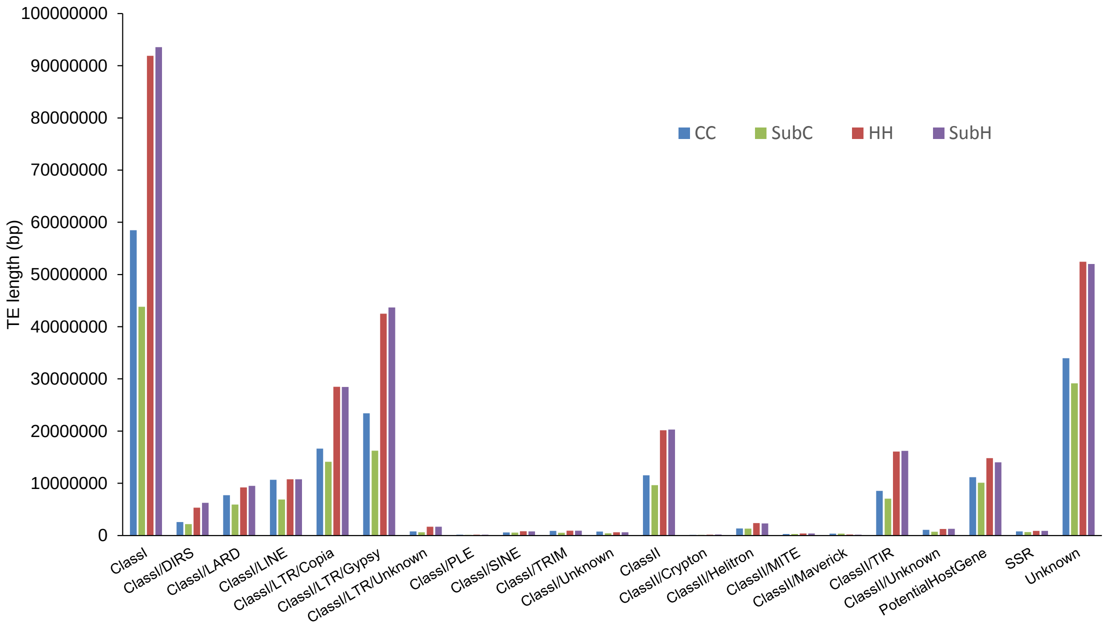

Figure S5. Comparison of the main transposable element (TE) types in syntenic region of the *C.* ×*hytivus* **(S_{14})** subgenomes and their parents *C. sativus* (CC) and *C. hystrix* (HH).

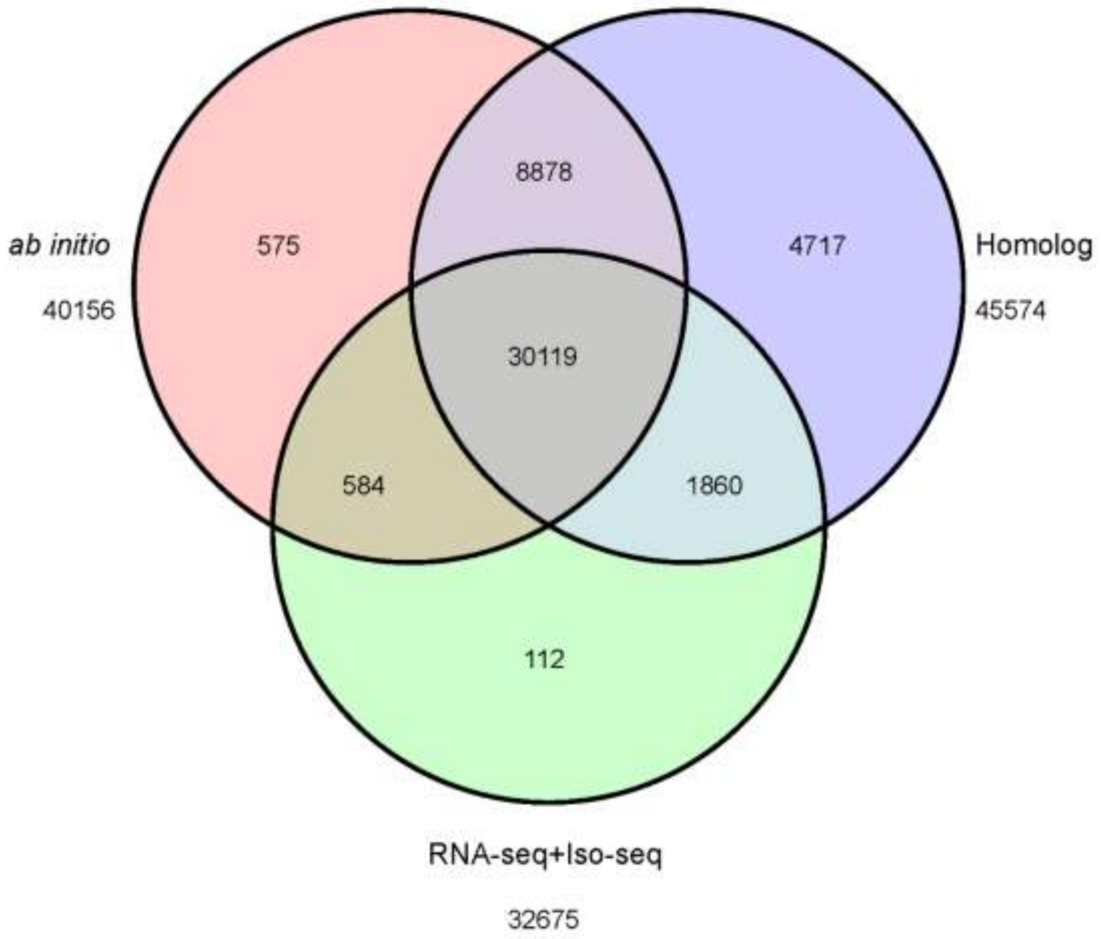

Figure S6. Venn diagram showing genes predicted by different methods. The numbers in the diagram representing the number of genes shared between or distinct to the indicated methods.

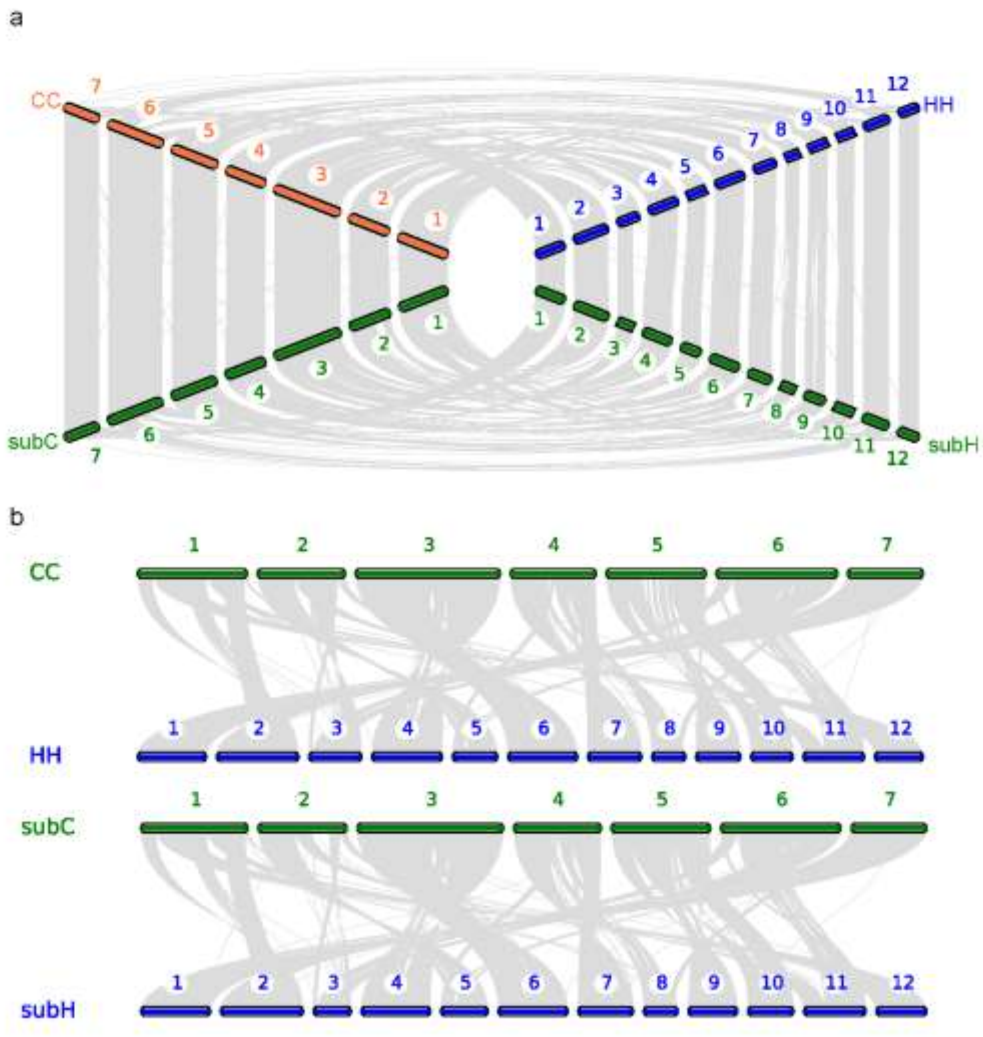

Figure S7. Syntenic comparisons among subgenomes of *C. ×hytivus* (S_{14}), *C. sativus* (CC), and *C. hystrix* (HH) genomes. a) Reciprocal syntenic comparison of *C. ×hytivus* (S_{14}) and its parental diploid genomes. b) Collinearity between the sub H- and C-genome in *C. ×hytivus* (S_{14}) and the HH and CC genome in diploid parents.

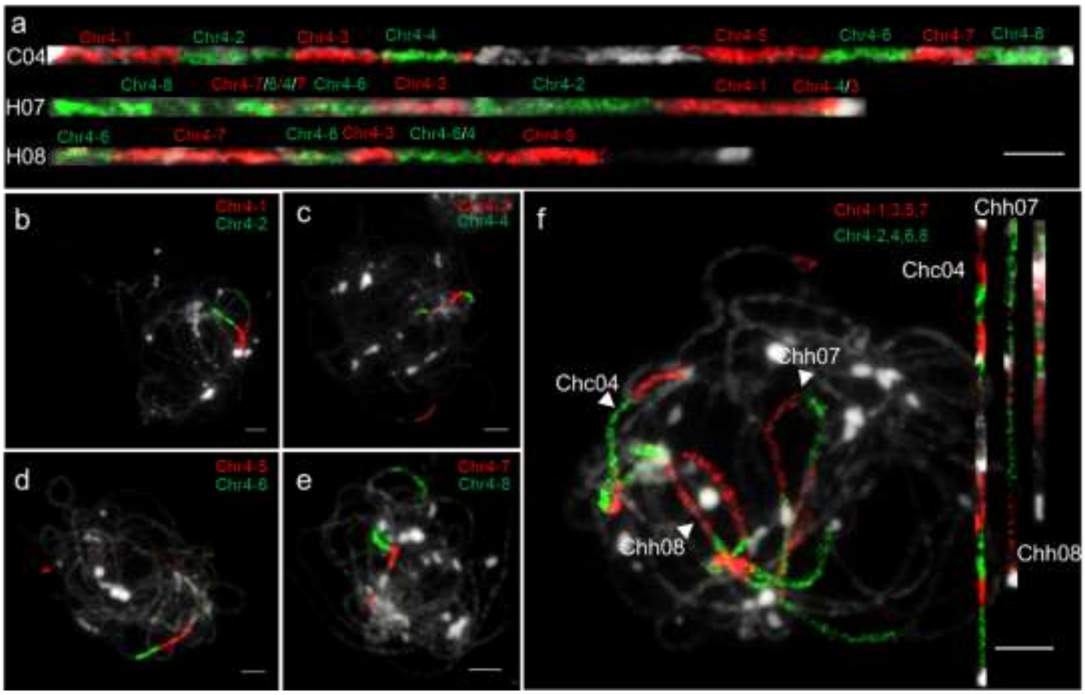

Figure S8. Comparative chromosome painting using 8 oligos sub-pools from cucumber Chr4. **a)** Syntenic blocks between *C. sativus* chromosome C04 and *C. hystrix* chromosomes H07 and H08. **b-e)** The painting was performed using every two subsections probes to accurately anchor each syntenic blocks: **b)** Chr4-1 (red) and Chr4-2 (green), **c)** Chr4-3 (red) and Chr4-4 (green), **d)** Chr4-5 (red) and Chr4-6 (green), **e)** Chr4-7 (red) and Chr4-8 (green). **f)** Chromosome segmentation painting on meiotic pachytene chromosomes of *C.* ×*hytivus* (S_{14}) using oligo probes of Chr4-1, -3, -5, -7 and Chr4-2, -4, -6, -8. Scale bars indicate 5 μm.

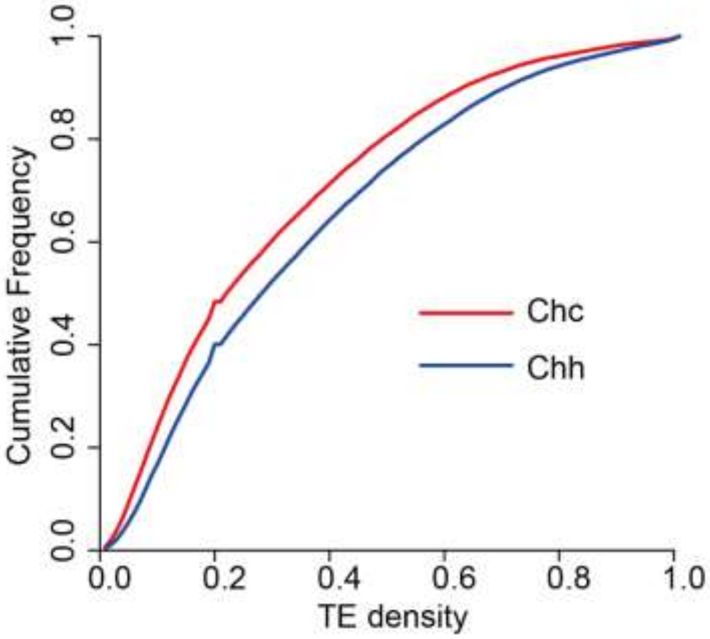

Figure S9. Comparison of TE densities near genes between the Chc and Chh subgenomes of *C. ×hytivus* (S_{14}).

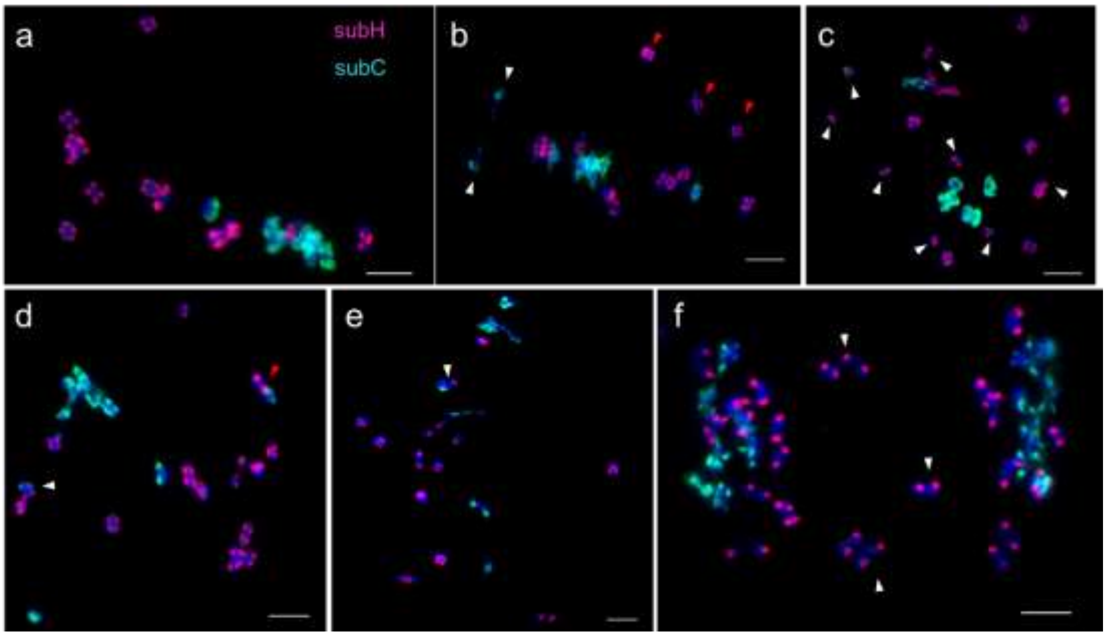

Figure S10. Representative abnormal meiotic chromosome behavior in PMCs of synthetic allotetraploid *C. ×hytivus*. **a)** GISH image shows 19 homologous bivalents, 12 H- bivalents (red) and 7 C- bivalents (green). **b)** One early disjunction C- bivalent (white arrows) and three H- bivalents that were not aligned to the equatorial plate (red arrows). **c)** Eight univalents from H-subgenome (white arrows). **d)** One C-H inter-genomic pairing (red arrow) and one HH-C trivalents (white arrow). **e)** One CC-H trivalents (white arrow). **f)** Five lagging H-subgenome chromosomes at anaphase I. Scale bars indicate 5 μm.

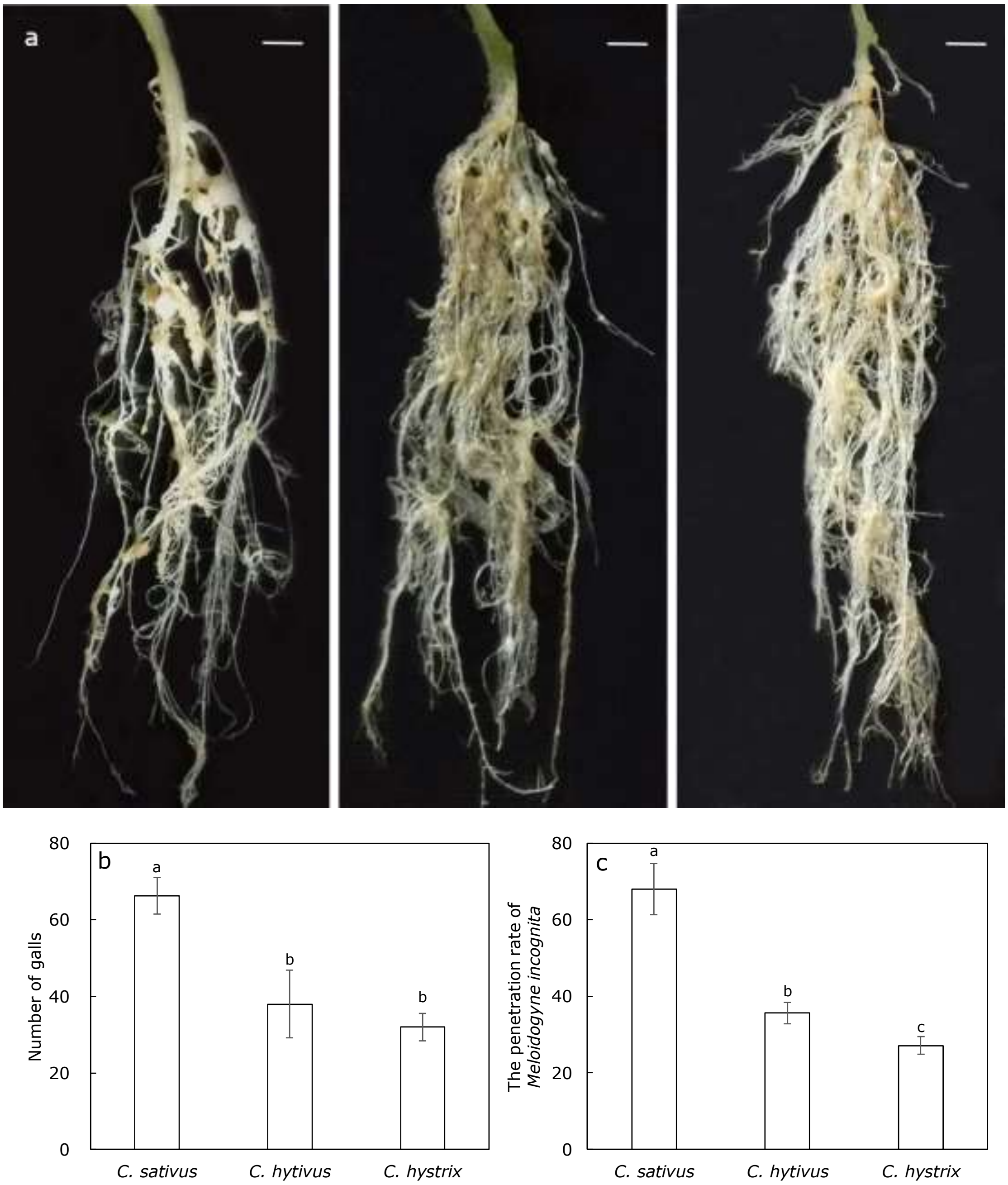

Figure S11. **a)** Root symptoms of *C. sativus* L. 'BejingJietou', *C.* ×*hytivus* (S_{14}) and *C. hystrix* Chakr. (from left to right) after 30 days inoculation with *Meloidogyne incognita*. **b)** Number of galls and **c)** the penetration rate of *Meloidogyne incognita* in *C. sativus* L. 'BejingJietou', *C.* ×*hytivus* (S_{14}), and *C. hystrix* Chakr. inoculated with *Meloidogyne incognita* for 30 days. Vertical bars represent the mean values ± SD (n = 4). Significance level $P < 0.05$. Scale bars indicate 1 cm.

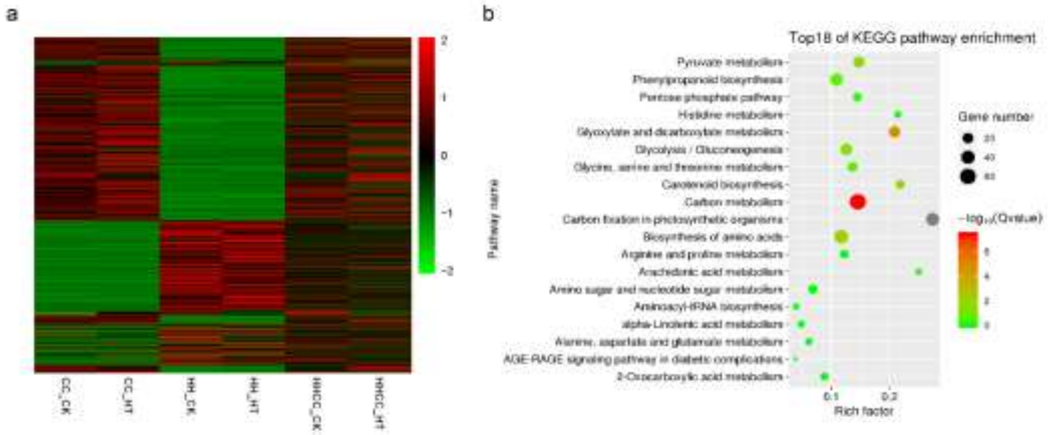

Figure S12. **a)** Heat maps of gene expression that altered after heat treatment exclusively in *C. ×hytivus* (S_{14}). **b** The KEGG (Kyoto Encyclopedia of Genes and Genomes, http://www.genome.jp/kegg) pathway enrichment analysis of these DEGs (differently expressed genes).

Table S1. Summary of BioNano data for *Cucumis* ×*hytivus*.

Information	
BioNano high quality data	
N mols	730,970
Total length (Gb)	163.4
Filtered (Gb)	108.46
(-minlen 150 -minsites 8 -maxIntensity 0.6)	
Average length (kb)	247.524
Mol N50 (kb)	245.221
Lab (/100kb)	9.617
Contig Coverage (×)	200
BioNano assembly results	
N Genome Maps	300
Total Length (Mb)	499.04
Mean Length (Mb)	1.66
Median Length (Mb)	1.03
L50 Length (Mb)	2.59
Maximum Length (Mb)	13.125

Table S2. Genome estimation by flow cytometry for *Cucumis* ×*hytivus* (S_{14}).

	Peak value	CV (%)	Genome size (Mb)
C. sativus (BeijingJietou) / *C.* ×*hytivus* (S_{14})	60.08 /141.60	10.00 /3.53	865.0
C. sativus (BeijingJietou) / *C.* ×*hytivus* (S_{14})	64.97 /141.00	8.45 /4.45	796.5
C. sativus (BeijingJietou) / *C.* ×*hytivus* (S_{14})	65.51 /134.31	8.73 /5.08	752.4
mean			804.6

Table S3. Summary of the predicted and assembled repeats in the genome of *Cucumis ×hytivus* (S_{14}).

Type	No. of reads	Percentage of the genome, %	Assembled length (bp)	Percentage of the genome, % (estimated genome size=699.87 bp)
C. hystrix 45S rRNA gene	2105381	1.8767	1347774	0.1926
C. sativus 45S rRNA gene	1046054	0.9324		
C. hystrix 5S rRNA gene	128041	0.1141	357912	0.0511
C. sativus 5S rRNA gene	53014	0.0473		
Type I/II	3200085	2.8524	1618847	0.2313
Type III	1545448	1.3776	2330073	0.3329
Type IV	4627569	4.1248	1364129	0.1949
Other predicted repeats	57610216	51.35	269717804	38.54
Total	70315808	62.6753	276736539	39.5428

Note: Other predicted repeats refer to predicted repeats exclude type I, II, III, IV satellite DNAs, 5S and 45S rDNA.

Table S4. Summary of Hi-C data for error correction and chromosome construction.

Library ID	H01
Insert size(bp)	350
Read pairs	188,164,110
Base number (bp)	56,320,109,636
Coverage (×)	104
Uniquely mapping pairs	109,378,897
Valid interaction pairs	64,534,262

Table S5. Hi-C assembly statistics.

Subgenome	Group	Sequence number	Sequence length (bp)
Chc	C01	35	34,248,536
	C02	46	28,288,356
	C03	36	43,400,534
	C04	103	35,111,878
	C05	44	34,444,263
	C06	28	33,018,195
	C07	44	23,132,997
Chh	H01	19	28,866,816
	H02	11	31,229,774
	H03	4	15,993,672
	H04	13	29,445,157
	H05	2	19,160,231
	H06	2	27,958,546
	H07	6	21,903,403
	H08	25	22,913,624
	H09	8	19,897,686
	H10	15	25,735,317
	H11	12	27,750,859
	H12	32	23,284,939
Total Sequences Clustered		485(73.04)	525,784,783(97.23)
Total Sequences Ordered and Oriented		121(24.95)	490,714,831(93.33)
Total Sequences unclustered		179	14,953,311
Total sequence		664	540,738,094

Table S6. Summary of repeat content in *Cucumis ×hytivus* (S_{14}), *C. sativus*, and *C. hystrix*. (excel)

Table S7. Summary of Iso-Seq. (excel)

Table S8. Statistics of gene prediction strategy.

Method	Software	Species	Gene number
Ab initio	Genscan	-	30,121
	Augustus	-	40,230
	GlimmerHMM	-	47,780
	GeneID	-	46,323
	SNAP	-	39,548
Homology-based	GeMoMa	*Arabidopsis thaliana*	35,614
		Oryza sativa	33,573
		Citrullus lanatus	42,160
		Cucumis melo	49,637
		Cucumis sativus	47,429
RNA-seq+ Iso-seq	PASA	-	74,619
	GeneMarkS-T	-	60,966
Integration	EVM	-	46,845

Table S9. Summary of predicted genes in *Cucumis ×hytivus* (S_{14}).

Category	Chc subgenome	Chh subgenome	Unknown	Total
Gene number	23108	22535	1202	46845
Gene length (bp)	85703136	93740821	2396341	181840298
Average gene length (bp)	3708.81	4159.79	1993.63	3881.74
Exon number	121855	121014	3324	246193
Exon length (bp)	34644735	36250203	868704	71763642
Average exon length (bp)	1499.25	1608.62	722.72	1531.94
CDS length per gene (bp)	1170.99	1187.17	686.76	1166.35
Exon num per gene	5.27	5.37	2.77	5.26
Intron number	98747	98479	2122	199348
Intron length (bp)	51058401	57490618	1527637	110076656
Average intron length (bp)	2209.56	2551.17	1270.91	2349.81
Intron number per gene	4.27	4.37	1.77	4.26

Table S10. Statistics of genes annotated by different databases.

Database	Total gene number	Percentage	Chc gene number	Percentage	Chh gene number	Percentage	Unknown gene number	Percentage
GO	22804	48.68%	11212	48.52%	11073	49.14%	519	43.18%
KEGG	15851	33.84%	7765	33.60%	7691	34.13%	395	32.86%
KOG	22421	47.86%	11037	47.76%	10965	48.66%	419	34.86%
TrEMBL	45549	97.23%	22715	98.30%	21749	96.51%	1085	90.27%
nr	45676	97.50%	22756	98.48%	21828	96.86%	1092	90.85%
Total	45687	97.53%	22757	98.48%	21832	96.88%	1098	91.35%

Table S11. Completeness inspection based on CEG and Plantae BUSCO database for *Cucumis ×hytivus* (S_{14}).

Information	
Complete BUSCOs	1309
Complete and single-copy BUSCOs	154
Complete and Duplicated BUSCOs	1155
Fragmented BUSCOs	34
Missing BUSCOs	97
Number of 458 CEGs present in assembly	448
% of 458 CEGs present in assemblies	97.82%
Number of 248 highly conserved CEGs present	224
% of 248 highly conserved CEGs present	90.32%

Table S12. Summary of non-coding RNAs and pseudogenes

Type		Number	Average Length (bp)	Total Length (bp)	% of Genome
miRNA		134	119.42	16002	0.003%
tRNA		1274	79.11	100782	0.019%
rRNA		2125	719.35	1528612	0.288%
	18S	403	1232.80	496820	0.094%
	28S	361	2397.07	865341	0.163%
	5.8S	258	142.39	36737	0.007%
	5S	1103	117.60	129717	0.024%
snRNA		573	110.52	63328	0.012%
	CD-box	306	101.53	31067	0.006%
	HACA-box	85	124.48	10581	0.002%
	Splicing	182	119.12	21680	0.004%
Pseudogenes		3439	1155.04	3972170	0.748%

Table S13. Summary of structural variant analysis.

	Initial SV number	Number of SVs verified with Illumina reads	Number of SVs excluded from HH assembly	Number of SVs excluded from CC assembly and genotype difference	Number of final true SVs	Number of final true SVs in the Chc subgenome	Number of final true SVs in the Chh subgenome
Translocation	9030	36	4	0	32	5	27
Transposition	14226	172	19	9	144	34	110
Deletion	23926	5432	346	395	4691	709	3982
Insertion	18001	2703	692	243	1768	447	1321
Inversion	7113	64	18	0	46	1	45

Table S14. Statistics of gene retention cases of the quartet genes between *Cucumis sativus* (CC genome), *C. hystrix* (HH genome), and *C.* ×*hytivus* (Chc and Chh subgenomes).

Cases	Number of gene pairs
CC-subC-HH-subH	15681
CC-subC-HH	874
CC-HH-subH	0
CC-subC-subH	1729
subC-HH-subH	363
CC-HH	266
CC-subC	1581
HH-subH	498
subC-subH	585
Total	21577

Table S15. Protein quartet table listing the homologous gene sets among CC (*Cucumis sativus*), HH (*C. hystrix*), Chc and Chh subgenomes of *C.* ×*hytivus*. A dot (.) is placed where no homolog is identified in the respective genome due to several possible causes (different annotation methods, gene loss, truncation, pseudogenization, matching random scaffolds, transposed). These changes are tracked in the next few columns labeled CC-status, HH-status, Chc-status, and Chh-status. (Excel)

Table S16. Detailed statistics of syntenic orthologs loss in one of the compared genomes/subgenomes

	Loss type	CC-subC	HH-subH
Syntenic blocks	Raw potential loss within syntenic blocks	266	1140
	Gene is predictable	115	206
	Partial loss	32	71
	Pseudogene	4	8
	Genes with depth >1 and coverage > 5%	115	853
	Gene loss within syntenic blocks	0	2
Synteny-excluded by blocks	Raw potential loss outside syntenic blocks	4186	6181
	Genes with depth >1 and coverage > 5%	4164	6037
	Gene loss outside syntenic blocks	22	144
Low depth and low coverage in actual male parent		11	
Final gene loss		11	146

Table S17. Overall list of *Cucumis ×hytivus* deleted genes with their putative function. (excel)

Table S18. CC to HH conversion regions inferred by both syntenic analysis and read coverage. (excel)

Table S19. HH to CC conversion regions inferred by both syntenic analysis and read coverage. (excel)

Table S20. Homeologous gene expression profile. (excel)

Table S21. Illumina reads statistics.

Sample ID	Library	BaseSum	GC(%)	Q20(%)	Q30(%)
F_1	270 bp	25,876,206,886	37.45	96.30	91.63
	3K	25,622,693,518	38.38	96.26	92.25
	4K	24,600,125,656	37.80	96.16	92.05
S_0	270 bp	22,658,914,564	37.88	96.26	91.59
	3K	30,862,422,132	37.90	95.85	91.53
	4K	21,953,990,556	37.14	96.40	92.49
S_4	270 bp	19,569,619,340	38.07	96.17	91.44
	3K	23,619,021,688	38.36	96.48	92.82

	4K	26,707,581,632	37.65	96.67	93.14
S_5	270 bp	21,159,909,070	37.44	96.10	91.45
	3K	26,160,424,250	39.05	94.75	89.99
	4K	24,304,640,652	37.76	94.75	89.94
S_6	270 bp	25,705,297,170	37.21	96.20	91.62
	3K	27,386,077,256	36.91	95.07	90.16
	4K	24,505,234,404	36.52	94.75	89.62
S_7	270 bp	24,394,700,068	37.23	95.97	91.26
	3K	20,011,708,104	38.20	95.33	90.63
	4K	19,670,409,682	37.82	95.02	90.01
S_8	270 bp	28,523,009,084	37.27	96.09	91.43
	3K	23,805,228,268	38.83	95.32	90.97
	4K	23,626,693,594	37.96	95.41	91.13
S_9	270 bp	24,694,180,048	37.41	96.10	91.47
	3K	24,091,959,996	38.70	95.17	90.74
	4K	20,263,719,894	38.22	95.06	90.38
S_{10}	270 bp	24,572,250,602	37.40	96.64	92.56
	3K	20,087,173,984	38.28	95.07	90.09
	4K	20,262,030,596	37.87	95.25	90.41
S_{11}	270 bp	24,581,743,442	37.17	96.69	92.68
	3K	23,820,475,538	38.29	96.52	92.87
	4K	23,495,452,914	37.66	96.61	93.01
S_{12}	270 bp	24,853,336,104	37.20	96.72	92.74
	3K	21,618,242,582	38.76	94.76	89.56
	4K	24,326,558,914	38.06	95.18	90.29
S_{13}	270 bp	27,868,124,042	37.10	96.74	92.78
	3K	24,757,592,582	38.06	95.15	90.25
	4K	21,583,616,480	37.76	95.06	90.08

Table S22. Genes confirmed deleted in *Cucumis ×hytivus* Chc subgenome and survey of their status in a diversity set of F_1, S_0, and subsequent generations (S_4-S_{13}). (excel)

Table S23. Genes confirmed deleted in *Cucumis ×hytivus* Chh subgenome and survey of their status in a diversity set of F_1, S_0, and subsequent generations (S_4-S_{13}). (excel)

Table S24. SNP detected in chloroplast genomes of F_1, S_0, and subsequent generations (S_4-S_{13}) of *Cucumis ×hytivus* in comparison to *C. hystrix*. (excel)

Table S25. Indels detected in chloroplast genomes of F_1, S_0, and subsequent generations (S_4-S_{13}) of *Cucumis ×hytivus* in comparison to *C. hystrix*. (excel)

Table S26. The structure and locus of R gene in *Cucumis ×hytivus*.

Table S27. Syntenic comparison of R gene in *Cucumis* ×*hytivus* and parents.

Table S28. KEGG enrichment list of genes having structural variant in the coding sequence or upstream region in *Cucumis* ×*hytivus*. (excel)

Table S29. Statistic of PacBio sub-reads length distribution.

Length (bp)	Num	Total length (bp)	Average length (bp)
0~2000	887,886	1,066,234,367	1,201
2000~4000	876,714	2,592,240,211	2,957
4000~6000	715,426	3,556,339,552	4,971
6000~8000	610,546	4,257,704,336	6,974
8000~10000	523,316	4,695,937,226	8,973
10000~12000	462,945	5,086,705,151	10,988
12000~14000	459,148	5,966,459,634	12,995
14000~16000	389,910	5,830,336,133	14,953
16000~18000	287,456	4,871,187,860	16,946
18000~	690,833	16,000,482,143	23,161
Total	5,904,180	53,923,626,613	9,133

Table S30. Genome assembly statistics of F_1, S_0, and subsequent generations (S_4-S_{13}) of *Cucumis* ×*hytivus*. (excel)
